# The signaling and selectivity of α‐adrenoceptor agonists for the human α2A, α2B and α2C‐adrenoceptors and comparison with human α1 and β‐adrenoceptors

**DOI:** 10.1002/prp2.1003

**Published:** 2022-09-13

**Authors:** Richard G. W. Proudman, Juliana Akinaga, Jillian G. Baker

**Affiliations:** ^1^ Cell Signalling Research Group, Division of Physiology, Pharmacology and Neuroscience, School of Life Sciences, C Floor Medical School, Queen's Medical Centre University of Nottingham Nottingham UK

**Keywords:** affinity, agonist, efficacy, hypertension, sedation, selectivity, α‐adrenoceptor

## Abstract

α2‐adrenoceptors, (α2A, α2B and α2C‐subtypes), are Gi‐coupled receptors. Central activation of brain α2A and α2C‐adrenoceptors is the main site for α2‐agonist mediated clinical responses in hypertension, ADHD, muscle spasm and ITU management of sedation, reduction in opiate requirements, nausea and delirium. However, despite having the same Gi‐potency in functional assays, some α2‐agonists also stimulate Gs‐responses whilst others do not. This was investigated. Agonist responses to 49 different α‐agonists were studied (CRE‐gene transcription, cAMP, ERK1/2‐phosphorylation and binding affinity) in CHO cells stably expressing the human α2A, α2B or α2C‐adrenoceptor, enabling ligand intrinsic efficacy to be determined (binding K_D_/Gi‐IC_50_). Ligands with high intrinsic efficacy (e.g., brimonidine and moxonidine at α2A) stimulated biphasic (Gi‐Gs) concentration responses, however for ligands with low intrinsic efficacy (e.g., naphazoline), responses were monophasic (Gi‐only). ERK1/2‐phosphorylation responses appeared to be Gi‐mediated. For Gs‐mediated responses to be observed, both a system with high receptor reserve and high agonist intrinsic efficacy were required. From the Gi‐mediated efficacy ratio, the degree of Gs‐coupling could be predicted. The clinical relevance and precise receptor conformational changes that occur, given the structural diversity of compounds with high intrinsic efficacy, remains to be determined. Comparison with α1 and β1/β2‐adrenoceptors demonstrated subclass affinity selectivity for some compounds (e.g., α2:dexmedetomidine, α1:A61603) whilst e.g., oxymetazoline had high affinity for both α2A and α1A‐subtypes, compared to all others. Some compounds had subclass selectivity due to selective intrinsic efficacy (e.g., α2:brimonidine, α1:methoxamine/etilefrine). A detailed knowledge of these agonist characteristics is vital for improving computer‐based deep‐learning and drug design.

## Graphical abstract

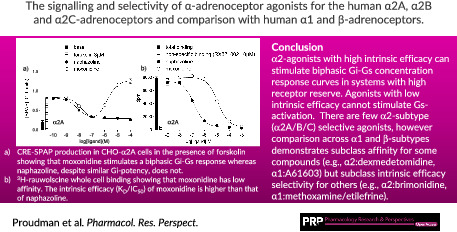

**Abbreviations**
CHO: Chinese hamster ovary
PBS: phosphate buffered saline
PDBU: phorbol 12,13‐dibutyrate
PTX: pertussis toxin
sfm: serum free media = DMEM/F12 containing 2 mM L‐glutamine

## 1 INTRODUCTION

α2‐adrenoceptors, comprising α2A, α2B and α2C‐subtypes, are Gi‐coupled G‐protein coupled receptors (GPCRs) expressed in heart, blood vessels and kidney (important for blood pressure^1^), but also on platelets and in brain.^2^, ^3^ Clonidine, the prototypical α2‐agonist developed in 1962 as a nasal decongestant/topical vasoconstrictor, caused unexpected bradycardia, hypotension and sedation (as noted by the trial physician who allowed his secretary to administer herself a few drops of nasal clonidine as she had a cold: she unexpectedly fell asleep for 24 h, and became bradycardic and hypotensive, but fully recovered), leading to the development of centrally‐acting α2‐agonist drugs.^3^, ^4^ Now, central activation of α2‐adrenoceptors is the main target for α2‐agonist antihypertensive drugs along with more recent α2‐adrenoceptor neurological and psychiatric modulation.^3^, ^5^, ^6^, ^7^ Central α2‐adrenoceptors include presynaptic autoreceptors, where noradrenaline activation inhibits further noradrenaline release from the same neuron, pre‐synaptic heteroreceptors where noradrenaline activation inhibits the release of other neurotransmitters, and postsynaptic receptors.^3^, ^5^, ^6^, ^7^, ^8^, ^9^ After clonidine, further α2‐agonists were developed with different properties, such as less lipophilic brimonidine (UK14304) aiming to reduce blood brain barrier transmission and sedation.^10^, ^11^ Brimonidine was also more efficacious, similar to adrenaline and noradrenaline, while clonidine had partial agonist activity.^12^, ^13^

In the brain, 90% of α2‐adrenoceptors are α2A‐adrenoceptors (as measured by receptor number not mRNA) and are highly expressed throughout, including the prefrontal cortex and locus coeruleus.^6^, ^14^, ^15^ Many physiological and pharmacological functions, and therefore targets for clinical α2‐agonists, are through activation of these α2A‐adrenoceptors.^2^, ^5^, ^15^ As well as antihypertensive properties, α2‐agonists are now used for sedation, to improve delirium, for ADHD, help with panic and pain, and to minimse withdrawal symptoms from opioids, benzodiazepines, alcohol and nicotine.^16^

A broad range of α2‐agonists exist with different pharmacological and physicochemical properties and clinical uses. Dexmedetomidine is one of the most potent α2‐agonists to date^17^ and is increasingly used in intensive care. It is used to sedate people requiring prolonged ventilation, induce short‐term sedation for procedures, as an adjunct to reduce doses of other sedatives (where a particular benefit is its lack of respiratory depression), reduce opiate consumption, reduce nausea and reduce delirium often seen post‐operatively and in intensive care patients.^16^, ^18^, ^19^ It also has potential to help with delirium, agitation and induce sedation in the palliative care setting.^19^ Furthermore, dexmedetomidine acts through endogenous sleep pathways,^20^ mimicking natural sleep and has a unique window for inducing “arousal” or “cooperative” sedation, enabling neurosurgery to be undertaken in awake patients.^18^, ^21^ Clonidine and guanfacine are used in ADHD patients and avoid the hypertensive and cardiovascular risks of the traditional stimulants methylphenidate and amphetamine.^7^ Tizanidine helps spasticity, muscle spasm and muscle cramps.^16^ Bromonidine and oxymetazoline are still used as topical vasoconstrictors in rosacea^22^ and brimonidine for glaucoma where it reduces aqueous humor production whilst increasing its outflow.^11^

The remaining 10% of brain α2‐adrenoceptors are α2C‐adrenoceptors and appear particularly prevalent in the striatum and hippocampus.^14^ The expression and effects of the α2B‐adrenoceptors appear very minor in brain.^6^

α2‐adrenoceptors have been extensively studied. The original studies were restricted to using different tissue preparations ‐ human platelet, colonic adenocarcinoma or rat cortex for α2A, neonatal rat lung for α2B and opossum kidney for α2C; e.g.,^23^, ^24^, ^25^ introducing problems of species variation. Other studies have shown that α2‐adrenoceptors couple to both Gi and Gs‐proteins and thus have a biphasic agonist concentration response – cAMP inhibition at low agonist concentrations followed by cAMP stimulation at high agonist concentrations.^17^, ^26^, ^27^, ^28^, ^29^, ^30^, ^31^, ^32^ However, for reasons unknown, only some compounds activate Gs‐stimulated cAMP while other compounds of similar Gi‐potency have no stimulatory response.^33^

Agonist drugs (and all drugs) have 2 important properties – affinity (ability to bind to a receptor) and intrinsic efficacy (ability to induce a response^34^, ^35^, ^36^, ^37^: a neutral antagonist having zero efficacy and thus only affinity to measure). An identical concentration response may result from a compound with high affinity and lower intrinsic efficacy, or a compound with low affinity but greater intrinsic efficacy. This property of intrinsic efficacy, as well as affinity may affect the selectivity of compounds^35^, ^38^ and underpin some the pharmacological heterogeneity seen between agonists.

This study measured the Gi and Gs‐coupled agonist responses and binding affinity of a wide range of α‐agonists in CHO cells expressing the human α2A, α2B or α2C‐adrenoceptor and investigated, then uncovered, the reason why some agonists induce Gs‐stimulation whilst others do not. Furthermore, as these measurements were determined using exactly the same technique in human β1 and β2‐adrenoceptors and α1‐adrenoceptors,^39^ this study provides a data set of the affinity, intrinsic efficacy and selectivity of ligands across the 8 most commonly targeted human adrenoceptors, measured under identical conditions.

## 2 METHODS

### 2.1 Materials

All compounds, together with the supplier and catalogue number are given in alphabetical order in Supplementary Data Table S1. ^3^H‐rauwolscine (a stereoisomer of yohimbine), ^3^H‐CGP12177, Microscint 20 and Ultima Gold XR scintillation fluid were from PerkinElmer (Buckinghamshire, UK). Foetal calf serum was from Gibco (Thermo‐Fisher), Lipofectamine and OPTIMEM were from Life Technologies, Thermo‐Fisher, Massachusetts USA. All other cell culture reagents were from Sigma Chemicals (Poole, Dorset, UK). Even though they are the same compound, brimonidine and UK14304 were purchased from different suppliers so are reported separately throughout. Medetomidine (racemate) and the active isomer dexmedetomidine were also purchased separately so reported separately.

### 2.2 Cell lines and cell culture

CHO‐K1 (RIDD: CVCL_0214) stably transfected with a CRE‐SPAP reporter gene and the human α2A‐adrenoceptor (CHO‐α2A), human α2B‐adrenoceptor (CHO‐α2B) or human α2C‐adrenoceptor (CHO‐α2C) were used^40^ as were lines expressing the same CRE‐SPAP reporter and human β1‐adrenoceptor (CHO‐β1) or human β2‐adrenoceptor (CHO‐β2,^38^). The parental cell line, which expresses the CRE‐SPAP reporter but no transfected receptor, and from which these lines were generated, was also used. All cells were grown in Dulbecco's modified Eagle's medium nutrient mix F12 (DMEM/F12) containing 10% foetal calf serum and 2 mM L‐glutamine in a 37°C humidified 5% CO_2_: 95% air atmosphere. Cells were always grown in the absence of any antibiotics. Mycoplasma contamination has intermittently been monitored within the laboratory (negative) but cell lines were not tested routinely with each experiment.

### 2.3 CRE‐SPAP gene transcription

CRE‐SPAP production was measured as in.^41^ Briefly, cells were grown to confluence in clear 96‐well plates in 100 μL DMEM/F12 containing 10% fetal calf serum and 2 mM L‐glutamine, and serum‐starved with serum free media (sfm, DMEM/F12 containing 2 mM L‐glutamine) 24 h before experimentation. Where used, pertussis toxin (PTX 100 ng/mL) was added to this sfm and thus the cells received 24 h treatment with PTX. On the experiment day, the sfm was removed and replaced with 100 μL sfm or 100 μL sfm containing antagonist at the final required concentration. Agonist in 10 μL (diluted in sfm) was then added to each well and the plates incubated at 37°C for 10 min, followed by 10 μM addition of forskolin (final well concentration 3 μM) and cells incubated for 5 h at 37°C (5% CO_2_). After 5 h, all drugs and media were removed, 40 μL sfm was added to each well and the cells incubated for a further hour at 37°C before being incubated at 65°C for 30 min (to destroy any endogenous phosphatases), cooled to 37°C, 100 μL 5 mM pNPP in diethanolamine buffer added to each well and incubated at 37°C until the yellow color developed before being read on a Dynatech MRX plate reader at 405 nm.

### 2.4 ^3^H‐cAMP accumulation

Cells were grown to confluence in 48‐well clear plates. Cells were pre‐labeled by incubation with 2 μCi/mL ^3^H‐adenine (0.5 mL per well) for 2 h at 37°C (5% CO_2_). The ^3^H‐adenine was removed, each well washed by the addition and removal of 1 mL sfm, then 0.5 mL sfm containing 100 μM IBMX added to each well. Agonist in 5 μL (diluted in sfm) was added to triplicate wells and incubated for 10 min at 37°C. Where used, forskolin (10 μM) was then added to the wells, and plates incubated for 5 h at 37°C (5% CO_2_). The reaction was terminated by the addition of 50 μL concentrated HCl per well, the plates were then frozen, thawed and ^3^H‐cAMP separated from other ^3^H‐nucleotides by Dowex and alumina column chromatography, with each column being corrected for efficiency by comparison with ^14^C‐cAMP recovery as previously described.^38^

### 2.5 ERK1/2‐phosphorylation

Extracellular‐signal‐regulated kinases (ERK1/2) activation was measured using a Surefire Alphascreen pERK1/2 kit. Cells were grown to confluence in 96‐well clear plates and double serum starved by washing the cells twice with 100 μL sfm before incubating in a further (third) 100 μL sfm for 24 h. Agonists in 20 μL sfm were added to the well (wells contained about 80 μL after some evaporation over 24 h, thus approximately a 1:5 dilution) and incubated for 2–4 min (at 37°C). Reagents were then removed, 20 μL lysis buffer added to each well and ERK1/2‐phosphorylation measured using the Alphascreen kit as per manufacturer's instructions. After a minimum of 2 h in the dark, the plates were read on an EnVision plate reader using standard Alphascreen settings. Basal and maximum ERK1/2‐phosphorylation (as determined by 10 μM PDBu, Phorbol 12,13‐dibutyrate) was measured in each plate.

### 2.6 ^3^H‐rauwolscine (yohimbine) whole cell binding

The affinity of the agonists was assessed using the whole cell binding and is identical to that used to determine the affinity of agonists at the α1‐adrenoceptors^39^ and β‐adrenoceptors.^38^ Cells were grown to confluence in white‐sided 96‐well plates. Media was removed from each well and 100 μL ligand (diluted in sfm to twice their final concentration) added to triplicate wells, followed immediately by the addition of 100 μL ^3^H‐rauwolscine (diluted in sfm) and incubated for 2 h at 37°C (5% CO_2_, humidified atmosphere). The media and all drugs were then removed from the wells, the cells washed twice by the addition and removed of 2 × 200 μL 4°C PBS. Cells were inspected under a light microscope to ensure they were still adherent after the wash, and 100 μL Microscint 20 was then added to each well. Total binding and non‐specific binding (determined by the presence of 10 μM RX821002) was defined in every plate. Radioligand concentrations were determined from taking the average of triplicate 50 μL samples of each ^3^H‐rauwolscine concentration used and counted on a PerkinElmer TriCarb Scintillation counter.

### 2.7 Data analysis

#### 2.7.1 Functional experiments—One‐site concentration responses curves

Many agonist responses were best described by a one‐site sigmoidal agonist concentration‐response curve. These were fitted to the data using the following equation with Graphpad Prism 7:

$$\text{Response}=\frac{\text{Emax}\times \left[A\right]}{{\mathrm{EC}}_{50}+\left[A\right]},$$

where Emax is the maximal response, [A] is the agonist concentration and EC_50_ is the concentration of agonist that produces 50% of the maximal response.

#### 2.7.2 Functional experiments—Two‐site concentration responses curves

Many concentration response curves clearly contained two components – an inhibitory response followed by a stimulatory response, thus a two‐site analysis was performed using the following equation:

$$\text{Response}=\text{Basal}+\left(\mathrm{FK}-\text{Basal}\right)\left[1-\frac{\left[A\right]}{\left(\left[A\right]+{\mathrm{IC}}_{50}\right)}\right]+S_{\mathrm{MAX}}\left[\frac{\left[A\right]}{\left(\left[A\right]+{\mathrm{EC}}_{50}\right)}\right],$$

where basal is the response in the absence of agonist, FK is the response to a fixed concentration of forskolin, [A] is the concentration of agonist, IC_50_ is the concentration of agonist that inhibits 50% of the response to forskolin (Gi‐coupled response), EC_50_ is the concentration of agonist that caused a half maximal stimulation (Gs‐coupled response) and S_MAX_ is the maximum stimulation of this Gs‐coupled‐component.

#### 2.7.3 Functional experiments—Calculation of antagonist K_D_ values from a parallel shift

Antagonist *K*_*D*_ values were calculated from the parallel shift of the agonist concentration responses in the presence of a fixed concentration of antagonist using the following equation:

$$\mathrm{DR}=1+\frac{\left[B\right]}{K_{D}},$$

where DR (dose ratio) is the ratio of the agonist concentration required to stimulate an identical response in the presence and absence of a fixed concentration of antagonist [B].

In experiments where three different fixed concentrations of the same antagonist were used, Schild plots were constructed using the following equation:

$$\mathrm{Log}\left(\mathrm{DR}-1\right)=\log\left[B\right]–\log\;\left(K_{D}\right).$$

A straight line was fitted to the points and a slope of 1 indicates competitive antagonism.^42^

#### 2.7.4 Calculation of agonist *K*_*D*_ from ^3^H‐rauwolscine whole cell competition binding

In all cases where a *K*_*D*_ value is stated, increasing concentrations of agonist fully inhibited the specific binding of ^3^H‐rauwolscine (unless otherwise annotated in the tables). The following equation was then fitted to the data using Graphpad Prism 7 and the IC_50_ was determined as the concentration required to inhibit 50% of the specific binding.

$$\%\text{specific binding}=100-\frac{\left(100\times \left[A\right]\right)}{\left(\left[A\right]+{\mathrm{IC}}_{50}\right)},$$

where [A] is the concentration of the competing agonist and IC_50_ is the concentration at which half of the specific binding of ^3^H‐rauwolscine has been inhibited.

From the IC_50_ value, the known concentration of ^3^H‐rauwolscine and the known *K*_*D*_^3^H‐rauwolscine (determined from saturation binding),^40^ a *K*_*D*_ value (concentration at which half the receptors are bound by the competing agonist ligand) was calculated using the Cheng‐Prusoff equation:

$$K_{D}\;\text{competing agonist}=\frac{{\mathrm{IC}}_{50}}{1+\left(\left[{}^{3}H‐\text{rauwolscine}\right]/{K_{D}}^{3}H‐\text{rauwolscine}\right)}.$$

In some cases the maximum concentration of competing ligand was not able to inhibit all of the specific ^3^H‐rauwolscine binding. Where no inhibition of radioligand binding was seen, even with maximum concentration of competing ligand possible, “no binding” is given in the tables. Where the inhibition produced by the maximum concentration of the competing ligand was 50% or less, an IC_50_ could not be determined and thus a K_D_ value not calculated. This is shown in the tables as IC_50_ > top concentration used (i.e. IC_50_ > 100 μM means that 100 μM inhibited some but less than 50% of the specific binding). In cases where the competing ligand caused a substantial (greater than 50%, but not 100%) inhibition of specific binding, an IC_50_ value was determined by extrapolating the curve to non‐specific levels and assuming that a greater concentration would have resulted in 100% inhibition. These values are given as apparent *K*_*D*_ values in the tables.

All data are presented as mean ± SEM of triplicate determinations and *n* in the text refers to the number of separate experiments. Affinity selectivity ratios are given as a ratio of the *K*_*D*_ values for the different receptors, and intrinsic efficacy is given as efficacy ratios determined from *K*_*D*_/IC_50_.^34^, ^36^, ^37^, ^43^

Key protein targets and ligands in this article are hyperlinked to corresponding entries in http://www.guidetopharmacology.org, the common portal for data from the IUPHAR/BPS Guide to PHARMACOLOGY,^44^ and are permanently archived in the Concise Guide to PHARMACOLOGY 2019/20.^45^

## 3 RESULTS

### 3.1 CHO‐a2A—Brimonidine

**FIGURE 1:**
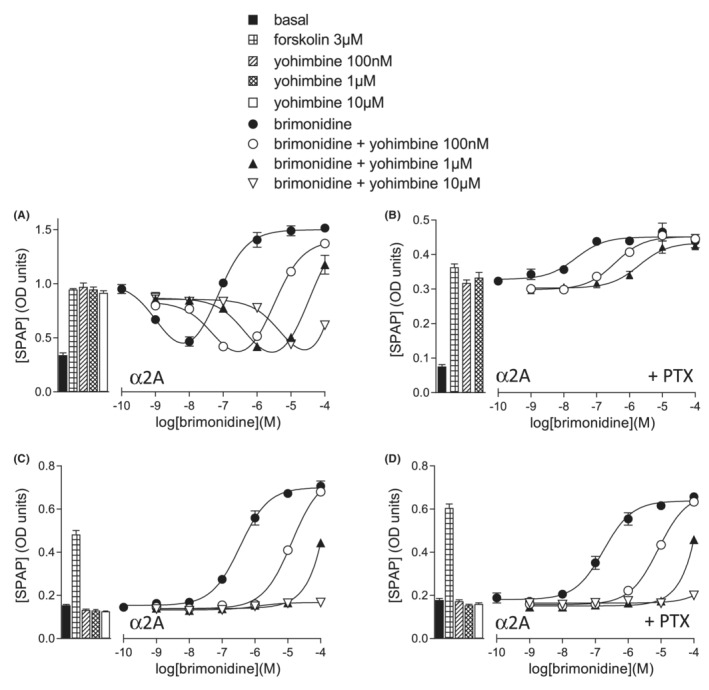
CRE‐SPAP in CHO‐α2A cells in response to brimonidine in the absence and presence of yohimbine. (A) in the presence of 3 μM forskolin, (B) in the presence of 3 μM forskolin after 24 h PTX pre‐treatment, (C) in the absence of forskolin and (D) in the absence of forskolin after 24 h PTX pre‐treatment. Bars represent basal CRE‐SPAP production, that in response to 3 μM forskolin alone, and that in response to yohimbine 100 nM, 1 μM and 10 μM alone. Data points are mean ± SEM of triplicate determinations. The Schild slopes are (a) 1.00 ± 0.08, *n* = 5 for inhibitory (Gi) component and 0.92 ± 0.11 *n* = 5 for stimulatory (Gs) component.

The α2‐adrenoceptors are predominantly Gi‐coupled receptors so inhibition of forskolin‐stimulated CRE‐SPAP production was initially evaluated. In CHO‐α2A cells, brimonidine stimulated a biphasic concentration response with an initial decrease of forskolin‐stimulated CRE‐SPAP production at low concentrations (log IC_50_–8.94 ± 0.05, *n* = 26), followed by a stimulation of CRE‐SPAP production at higher concentrations (log EC_50_–7.07 ± 0.04, *n* = 26; Figure 1A; Table 1). Pre‐treatment with PTX (which inactivates Gi‐proteins by ADP‐ribosylation^46^ and had no effect on the baseline or forskolin‐stimulated control measurements), abolished the inhibitory response but left the stimulatory responses intact (EC_50_–7.81 ± 0.06, 1.33 ± 0.03 fold increase, *n* = 11; Figure 1B). This suggests that the initial inhibitory response is occurring via Gi‐coupling and the stimulatory response via Gs‐coupling. When examined in the absence of forskolin, the stimulatory (Gs‐coupled) response of brimonidine remained (log EC_50_–6.67 ± 0.06, 160.8 ± 9.6% of the response to 3 μM forskolin, *n* = 11; Figure 1C,D).

**TABLE 1:** Data obtained in CHO‐α2A cells. Log K_D_ values from ^3^H‐rauwolscine whole cell binding (see Table 4 for mean ± sem and n numbers); biphasic log IC_50_ and EC_50_ values from CRE‐SPAP production in presence of forskolin, or in the cases of inhibition only, log IC_50_ and % inhibition from the 3 μM forskolin control; log efficacy ratio (K_D_/IC_50_); log EC_50_ and % maximum response compared to 3 μM forskolin from CRE‐SPAP production in the absence of forskolin; and log EC_50_ and % maximum response compared to 10 μM PDBU from ERK1/2‐phosphorylation. The ligands are arranged in order of α2A intrinsic efficacy ratio (K_D_/IC_50_)

CHO‐α2A	binding		CRE‐SPAP (with forskolin)				Log efficacy ratio	CRE‐SPAP (without forskolin)			ERK1/2‐phosphorylation		
	Log K_D_	*n*	Log IC_50_ (Gi)	Log EC_50_ (Gs)	% inhibition	*n*		Log EC_50_ (Gs)	% 3 μM forskolin	*n*	Log EC_50_	% 10 μM PDBu	*n*
Noradrenaline	−3.57	9	−6.60 ± 0.12	−5.29 ± 0.10		12	3.03	−5.16 ± 0.06	171.5 ± 11.1	6	−7.74 ± 0.18	121.9 ± 6.3	7
A61603	IC_50_ ~ 100 μM	5	−6.95 ± 0.06	−5.66 ± 0.13		10	>2.95	−5.48 ± 0.33	5.9 ± 2.2	7	−7.99 ± 0.11	151.1 ± 14.5	6
α‐methylnorepinephrine	−3.69	5	−6.47 ± 0.05	−5.31 ± 0.05		13	2.78	−5.29 ± 0.02	171.1 ± 4.3	10	−7.82 ± 0.12	142.6 ± 22.3	7
Adrenaline	−3.74	10	−6.51 ± 0.10	−5.51 ± 0.05		12	2.77	−5.65 ± 0.10	203.0 ± 12.5	6	−7.95 ± 0.15	137.1 ± 15.3	7
UK14304	−6.41	5	−9.11 ± 0.09	−7.20 ± 0.05		8	2.70	−6.66 ± 0.06	167.4 ± 11.0	8	−9.41 ± 0.24	128.0 ± 11.3	5
Brimonidine	−6.37	5	−8.94 ± 0.05	−7.07 ± 0.04		26	2.57	−6.67 ± 0.06	160.8 ± 9.6	11	−9.14 ± 0.08	153.0 ± 12.5	7
Moxonidine	−5.02	5	−7.51 ± 0.07	−5.81 ± 0.03		10	2.49	−5.36 ± 0.02	164.2 ± 17.9	5	−8.52 ± 0.08	129.2 ± 6.0	6
Para‐amino‐clonidine	−6.35	5	−8.74 ± 0.12	−6.81 ± 0.15		8	2.39	−6.55 ± 0.10	37.6 ± 4.0	12	−9.58 ± 0.12	141.9 ± 10.6	7
Dopamine	−3.39	5	−5.44 ± 0.05	−4.09 ± 0.06		6	2.05	1 mM	47.8 ± 8.1	6	−6.77 ± 0.15	108.7 ± 13.0	6
Medetomidine	−7.52	5	−9.43 ± 0.09	−7.39 ± 0.09		6	1.91	−7.13 ± 4.7	23.1 ± 4.7	6	−9.68 ± 0.15	114.1 ± 9.3	7
RWJ52353	−4.76	5	−6.59 ± 0.04		90.6 ± 4.1	5	1.83	No response		5	−7.81 ± 0.08	130.2 ± 12.4	5
Tizanidine	−5.97	5	−7.59 ± 0.08	−5.82 ± 0.19		5	1.62	−5.85 ± 0.17	15.8 ± 4.7	7	−8.42 ± 0.14	135.7 ± 17.3	6
Isoprenaline	IC_50_ > −1 mM	5	−4.61 ± 0.10	~1 mM		5	>1.61	1 mM	17.1 ± 6.4	5	−6.00 ± 0.23	118.7 ± 24.1	6
Xylazine	−4.94	5	−6.54 ± 0.07	−5.07 ± 0.06		10	1.60	No response		5	−7.52 ± 0.14	128.3 ± 15.7	6
Dexmedetonidine	−7.70	6	−9.27 ± 0.09	−7.55 ± 0.08		14	1.57	−7.36 ± 0.08	23.6 ± 2.7	11	−9.54 ± 0.14	138.5 ± 15.4	7
Guanabenz	−6.96	6	−8.44 ± 0.07		77.7 ± 2.2	19	1.48	No response		5	−9.10 ± 0.10	134.3 ± 25.2	6
Clonidine	−6.72	5	−8.18 ± 0.04	−6.35 ± 0.12		20	1.46		<5%	9	−8.99 ± 0.12	137.7 ± 7.3	6
BHT920	−5.94	5	−7.40 ± 0.02	−5.87 ± 0.07		5	1.46	−5.59 ± 0.12	7.6 ± 2.4	7	−8.45 ± 0.06	125.8 ± 9.4	6
ST‐91	−6.15	6	−7.58 ± 0.06	−6.27 ± 0.16		5	1.43	No response		5	−8.52 ± 0.10	128.2 ± 17.5	6
Guanfacine	−6.58	6	−7.96 ± 0.11	−6.53 ± 0.10		10	1.38	No response		5	−8.95 ± 0.15	129.7 ± 13.4	7
BHT933	−4.89	5	−6.25 ± 0.08	−4.50 ± 0.10		5	1.36	No response		7	−7.20 ± 0.09	124.5 ± 12.6	6
Amitraz	−6.13	5	−7.38 ± 0.10		86.9 ± 2.3	7	1.25	No response		5	−7.75 ± 0.11	126.7 ± 12.0	7
Metaraminol	−4.28	5	−5.50 ± 0.12		87.0 ± 1.3	5	1.22	No response		5	−6.62 ± 0.14	136.4 ± 13.9	7
R‐phenylephrine	−4.89	5	−6.10 ± 0.07		86.9 ± 1.5	5	1.21	No response		6	−7.04 ± 0.10	127.8 ± 10.2	6
Tetrahydrozoline	−6.49	6	−7.67 ± 0.09		69.3 ± 7.2	5	1.18	No response		5	−8.44 ± 0.12	123.0 ± 12.8	6
Oxymetazoline	−7.27	11	−8.40 ± 0.07		81.0 ± 1.7	15	1.13	No response		5	#		
Detomidine	−7.41	5	−8.39 ± 0.07		82.6 ± 3.6	10	0.98	No response		6	−9.03 ± 0.08	133.3 ± 9.8	7
Chloroethylclonidine	−5.47	5	−6.45 ± 0.02		90.3 ± 2.5	5	0.98	No response		5	−6.69 ± 0.08	109.8 ± 17.2	8
Synephrine	−4.05	5	−5.02 ± 0.16		75.6 ± 5.6	7	0.97	No response		5	−6.11 ± 0.09	114.5 ± 12.3	6
Rilmenidine	−5.81	5	−6.77 ± 0.09		94.2 ± 3.6	5	0.96	No response		5	−7.83 ± 0.17	144.0 ± 22.0	7
Naphazoline	−7.01	5	−7.79 ± 0.07		83.1 ± 3.6	16	0.78	No response		5	−8.72 ± 0.15	118.1 ± 9.0	7
Etilefrine	−3.71	5	−4.32 ± 0.09		101.3 ± 4.6	7	0.61	No response		5	−5.49 ± 0.12	150.8 ± 10.2	6
Xylometazoline	−7.62	6	−8.13 ± 0.04		73.9 ± 4.6	5	0.51	No response		5	#		
Octopamine	−3.38	5	−3.88 ± 0.12		98.1 ± 5.7	5	0.50	No response		5	−5.32 ± 0.08	124.1 ± 11.7	6
Bromocriptine	−8.25	5	−8.28 ± 0.15		58.1 ± 3.8	5	0.03	No response		5	−9.14 ± 0.08^a^	105.8 ± 10.6	7
Allyphenyline	−6.92	5	−6.79 ± 0.21		50.6 ± 6.3	7	−0.13	No response		5	−7.82 ± 0.11	127.1 ± 13.0	6
Cirazoline	−6.38	5	−6.22 ± 0.13		43.6 ± 4.4	10	−0.16	No response		10	−6.80 ± 0.10	113.0 ± 14.3	6
Methoxamine	−4.03	5	IC_50_ > 100 μM			6		No response		5	−5.22 ± 0.14	120.9 ± 29.4	7
Dihydroergotamine	−8.59	5	No response			5		No response		5	#		
Atipamezole	−8.50	5	No response			5		No response		5	−7.54 ± 0.13	42.4 ± 5.9	6
Buspirone	−5.24	5	No response			7		No response		5	−5.44 ± 0.17	16.5 ± 3.8	6
Dobutamine	−4.69	5	No response			5		No response		5	−5.70 ± 0.12	115.2 ± 7.6	5
Ephedrine	−4.46	5	No response			5		No response		5	−4.78 ± 0.16	123.1 ± 17.3	7
T‐CG 1000	−7.08	5	No response			5		No response		5	−7.28 ± 0.07	79.0 ± 11.5	8
Salmeterol	−4.76	5	No response			5		No response		5			
Fenoterol	−3.46	5	No response			7		No response		6			
Formoterol	IC_50_ > 100 μM	5	No response			7		No response		6			
Midodrine	IC_50_ > 1 mM	5	No response			5		No response		5	100 μM	101.0 ± 15.5	9
Salbutamol	IC_50_ > 1 mM	5	No response			5		No response		5			

*Note*: # these compounds stimulate ERK1/2‐phosphorylation in parent CHO cells^39^ so measurements were not made in this cell line.

^a^ Bromocriptine also stimulated a response in parent CHO cells (see results, log EC_50_–6.93) but as this is far less potent that the response in CHO‐α2A cells (log EC_50_–9.14), it is included here as the CHO‐α2A response is likely to be α2A‐receptor mediated.

**FIGURE 2:**
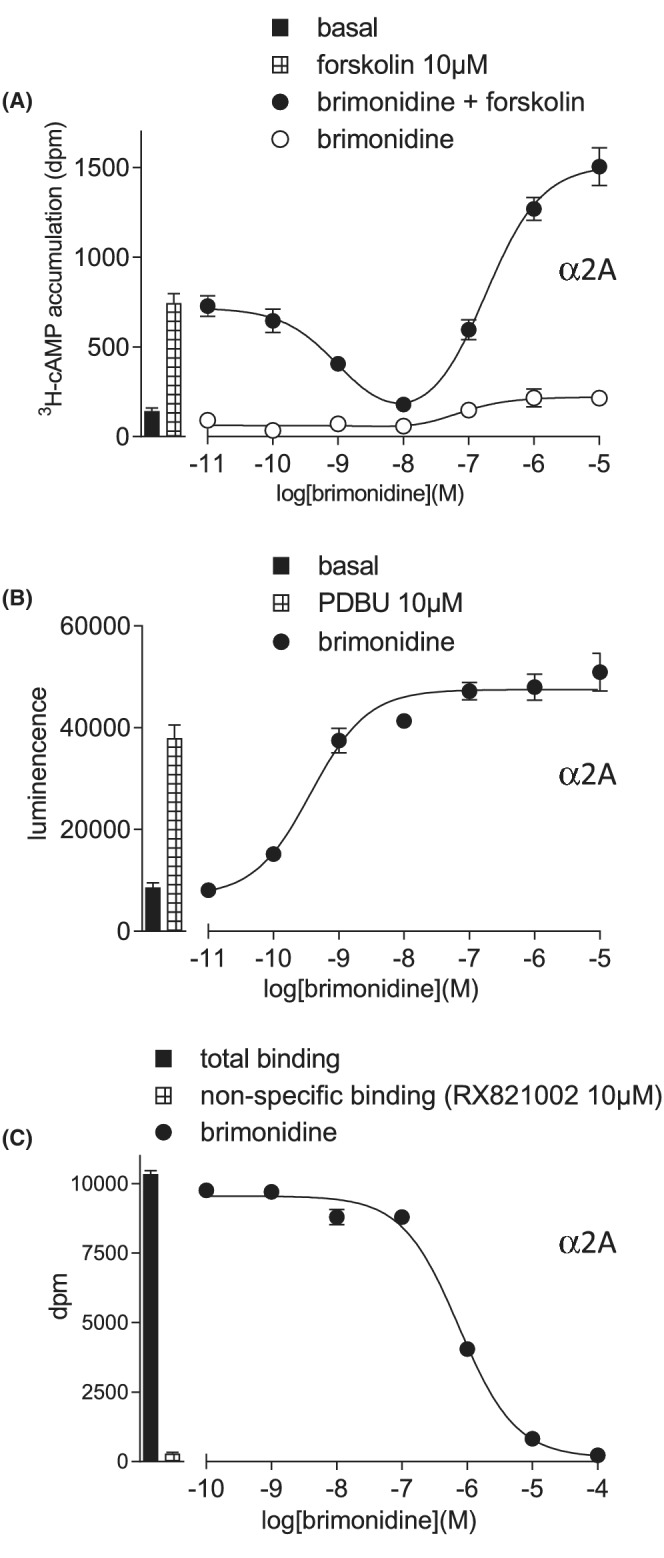
Responses to brimonidine in CHO‐α2A cells (A) ^3^H‐cAMP accumulation in the absence and presence of 10 μM forskolin. Bars represent basal ^3^H‐cAMP accumulation and that in response to 10 μM forskolin. (B) ERK1/2‐phosphorylation. Bars represent basal ERK1/2‐phosphorylation and that in response to 10 μM PDBu. (C) inhibition of ^3^H‐rauwolscine binding. Bars represent total binding and non‐specific binding as determined by 10 μM RX821002. The concentration of ^3^H‐rauwolscine in this experiment was 0.62 nM. Data points are mean ± SEM of triplicate determinations in all cases.

To confirm that CRE‐SPAP production was an accurate reflection of cAMP responses, direct cAMP measurements were made. Brimonidine stimulated a biphasic response in the presence of forskolin (log IC_50−_9.21 ± 0.10, log EC_50_–6.74 ± 0.09, *n* = 7), and stimulatory response in the absence of forskolin (log EC_50_–6.67 ± 0.12, 33.0 ± 4.5% forskolin 10 μM, n = 6), very similar to the CRE‐SPAP responses (Figure 2A). This is very similar to the biphasic cAMP response previously reported for α2A‐adrenopceptor expressed in CHO or HEK cells with adrenaline, noradrenaline, brimonidine, clonidine and guanabenz^17^, ^26^, ^27^, ^29^, ^30^, ^31^, ^32^, ^47^ and for a CRE‐reporter gene study in guinea pig α2A, α2B and α2C‐adrenoceptors.^28^

To confirm that both parts of these responses were occurring via the α2A‐adrenoceptor, the α2‐selective antagonist yohimbine was used to inhibit the response. Increasing concentrations of yohimbine caused a rightward shift of both the inhibitory (yohimbine log *K*_*D*_ −8.45 ± 0.03, *n* = 15; schild slope 1.00 ± 0.08, *n* = 5) and the stimulatory brimonidine response (yohimbine log *K*_*D*_ −8.65 ± 0.04, *n* = 13, schild slope 0.92 ± 0.11, *n* = 5; Figure 1A), as in.^26^ This affinity is similar to the affinity obtained for yohimbine from whole cell binding in these cells (log *K*_*D*_ −8.48).^40^ A similar high affinity for yohimbine was seen with the stimulatory brimonidine response in the presence of PTX (yohimbine log *K*_*D*_ −8.48 ± 0.13, *n* = 15; Figure 1B), and in the absence of forskolin (whether that be without PTX, Figure 1C, −8.61 ± 0.06, *n* = 14 or in the presence of PTX (Figure 1D, −8.54 ± 0.04, *n* = 12). Finally no response was seen to brimonidine in cells without the transfected receptor (see later).

### 3.2 Brimonidine response in α2A cells lines with different levels of receptor expression

To examine this biphasic response further, two other cell lines stably expressing the human α2A‐adrenoceptor at lower receptor expression levels were examined. As expected, lower receptor expression resulted in a rightward shift of the Gi‐coupled inhibitory brimonidine response (and for para‐amino‐clonidine, clonidine and naphazoline), however, there was a direct relationship between the receptor expression level and the ability to induce a Gs‐stimulatory response (both in the presence and absence of forskolin). As shown in supplementary Figure S1, in the presence of forskolin, as well as brimonidine Gi‐inhibition, cell line 1 (main CHO‐α2A cells used in this study with α2‐adrenoceptor expression level of 5830 fmol/mg protein) resulted in a large stimulatory component, to a level above that of the 3 μM forskolin stimulation, cell line 2 (expression level 4724 fmol/mg protein) resulted in less of a stimulatory component, reaching the level of the 3 μM forskolin stimulation, whilst cell line 3 (receptor expression level 121 fmoL/mg protein) had no Gs‐stimulatory response at all. This was also true in the absence of forskolin, where the brimonidine response in cell line 1 was 160.8% of the 3 μM forskolin response, less in cell line 2 (56.1%) and no response was seen in cell line 3. Thus the ability to stimulate a Gs‐coupled response at the α2A‐adrenoceptor is directly related to the receptor reserve within that system.

### 3.3 CHO‐α2A cells—Other α2‐agonists

**FIGURE 3:**
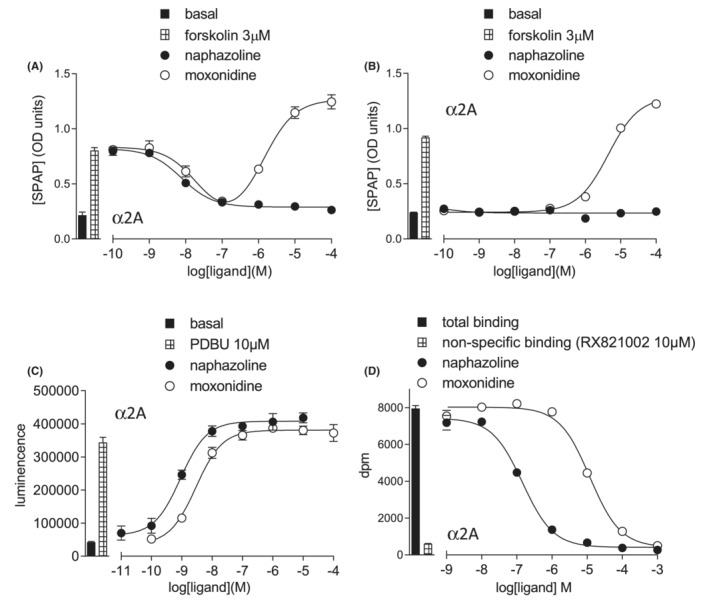
Responses to naphazoline and moxonidine in CHO‐α2A cells. (A) CRE‐SPAP production in the presence of 3 μM forskolin and (B) CRE‐SPAP production in the absence of forskolin. Bars respresent basal CRE‐SPAP production and that in response to 3 μM forskolin. (C) ERK1/2‐phosphorylation. Bars represent basal ERK1/2‐phosphorylation and that in response to 10 μM PDBu and (D) inhibition of ^3^H‐rauwolscine binding. The concentration of ^3^H‐ rauwolscine was 0.60 nM. Data points are mean ± SEM of triplicate determinations in all cases.

Not all agonists stimulated a biphasic response. Moxonidine stimulated a clear biphasic CRE‐SPAP production response, whilst naphazoline, despite a similar potency for the Gi‐component, did not (Figure 3A). In the absence of forskolin, moxonidine stimulated an agonist response whereas naphazoline did not (Figure 3B). Furthermore, examining many ligands showed that the ability to stimulate the Gs‐response was not an all or nothing event, but compounds exist with a graded range in the size of Gs‐mediated responses (Table 1). For example, dexmedetomidine, used increasingly in ITU, was able to simulate Gs‐coupling, however this was significantly less than that seen for brimonidine and the endogenous catecholamines (Supplementary Figure S2), whereas the Gs‐coupled response for clonidine was barely measureable.

### 3.4 CHO‐α2A‐ERK1/2 phosphorylation responses

When other responses were examined, brimonidine stimulated a potent ERK1/2‐phosphorylation response, with an EC_50_ (log EC_50_–9.14 ± 0.08, *n* = 7, Figure 2B) similar to that seen for the Gi‐coupled response. The responses to all agonists studied closely mirrored that of the Gi‐inhibitory CRE‐SPAP response (Table 1).

### 3.5 ^3^H‐rauwolscine whole cell binding and intrinsic efficacy ratio

Affinity measurements were made from ^3^H‐rauwolscine binding using the same media and conditions as for the functional assays (living cells). From the *K*_*D*_ values obtained and the IC_50_ value from the Gi‐inhibition of CRE‐SPAP production, an efficacy ratio (*K*_*D*_/IC_50_)^34^, ^36^, ^37^, ^43^ was obtained as a measure of the intrinsic efficacy of the agonist. This is the same analysis as^13^'s visual comparison in human fat cells where the clonidine concentration response from binding and lipolysis were superimposable, but the lipolysis response to adrenaline and brimonidine were left‐shifted with respect to binding, demonstrating greater intrinsic efficacy for adrenaline and brimonidine than clonidine. Thus efficacy ratios allow a numerical comparison and is a more accurate measure of true ligand intrinsic efficacy than either potency or maximal response.^48^ The affinity of brimonidine was relatively low (log *K*_*D*_ −6.37 ± 0.07, *n* = 5, Figure 2C;  Table 1), compared to its IC_50_ (−8.94) giving an intrinsic efficacy ratio of 2.57. This was similar for moxonidine (2.49). However, the efficacy ratio for naphazoline was only 0.78. The ligands in Table 1 (CHO‐α2A cells) are presented in order of decreasing efficacy ratio, as determined from Gi‐inhibition of CRE‐SPAP production and *K*_*D*_ from binding. However given the close correlation between IC_50_ and ERK1/2‐phosphorylation EC_50_, similar results would have occurred from using efficacy ratio calculated using the ERK1/2‐phosphorylation as the functional response.

### 3.6 CHO‐α2B cells

**FIGURE 4:**
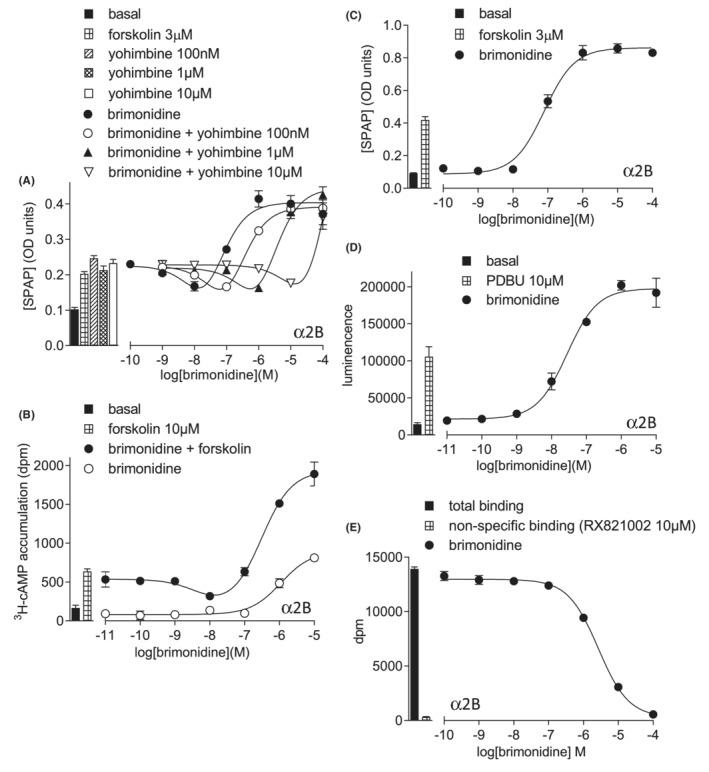
Responses to brimonidine in CHO‐α2B cells. (A) CRE‐SPAP production in the presence of 3 μM forskolin, in the presence and absence of yohimbine. Bars represent basal CRE‐SPAP production, that in response to 3 μM forskolin alone, and that in response to yohimbine 100 nM, 1 μM and 10 μM alone. (B) ^3^H‐cAMP accumulation in response to brimonidine in the absence and presence of 10 μM forskolin. Bars represent basal ^3^H‐cAMP accumulation and that in response to 10 μM forskolin. (C) CRE‐SPAP production in the absence of forskolin. Bars represent basal CRE‐SPAP production and that in response to 3 μM forskolin. (D) ERK1/2‐phosphorylation in response to brimonidine. Bars represent basal ERK1/2‐phosphorylation and that in response to 10 μM PDBu. and (E) inhibition of ^3^H‐rauwolscine binding in whole CHO‐α2B cells in response to brimonidine. Bars represent total binding and non‐specific binding as determined by 10 μM RX821002. The concentration of ^3^H‐rauwolscine in this experiment was 0.86 nM. Data points are mean ± SEM of triplicate determinations in all cases.

Brimonidine also stimulated a biphasic response in CHO‐α2B cells (Table 2). Both inhibitory and stimulatory parts of the response were inhibited by yohimbine to yield *K*_*D*_ values of −7.62 ± 0.14 and −7.66 ± 0.03 respectively (*n* = 8; Figure 4A), very similar to that obtained from whole cell binding (log *K*_*D*_ −7.66).^40^ As expected, Gs‐stimulatory responses were seen in the absence of forskolin (Figure 4B). Similar responses were also obtained from cAMP accumulation in the presence (log IC_50_ −8.19 ± 0.11, log EC_50_ −6.56 ± 0.08, *n* = 7) and absence (log EC_50_ −6.09 ± 0.11, 163.0 ± 15.2% 10 μM forskolin, *n* = 7) of forskolin and the ERK1/2‐phosphorylation response closely resembled the IC_50_ obtained from Gi‐inhibition (log −7.78, Table 2; Figure 4D).

**TABLE 2:** Data obtained in CHO‐α2B cells. Log K_D_ values from ^3^H‐rauwolscine whole cell binding (see Table 4 for mean ± SEM and n numbers); biphasic log IC_50_ and EC_50_ values from CRE‐SPAP production in presence of forskolin, or in the cases of inhibition only, log IC_50_ and % inhibition from the 3 μM forskolin control; log efficacy ratio (K_D_/IC_50_); log EC_50_ and % maximum response compared to 3 μM forskolin from CRE‐SPAP production in the absence of forskolin; and log EC_50_ and % maximum response compared to 10 μM PDBU from ERK1/2‐phosphorylation. The ligands are arranged in order of α2B intrinsic efficacy ratio (K_D_/IC_50_)

CHO‐α2B	binding		CRE‐SPAP (with forskolin)						CRE‐SPAP (without forskolin)			ERK1/2‐phosphorylation		
	Log K_D_	*n*	Log IC_50_ (Gi)	Log EC_50_ (Gs)	% inhibition	*n*	Log efficacy ratio		Log EC_50_ (Gs)	% 3 μM forskolin	*n*	Log EC_50_	% 10 μM PDBu	*n*
Noradrenaline	−3.52	9	−7.79 ± 0.15	−6.90 ± 0.14		11	4.27		−6.79 ± 0.07	194.5 ± 19.1	6	−7.81 ± 0.13	215.0 ± 32.0	6
α‐methylnorepin ephrine	−3.80	5	−7.94 ± 0.18	−6.99 ± 0.24		10	4.14		−7.54 ± 0.08	219.0 ± 49.4	3	−7.93 ± 0.19	192.6 ± 27.4	7
Adrenaline	−3.56	9	−7.64 ± 0.18	−6.27 ± 0.16		11	4.08		−6.64 ± 0.07	187.3 ± 11.6	7	−7.53 ± 0.12	175.8 ± 20.7	7
Metaraminol	−4.11	8	−8.14 ± 0.14	−7.09 ± 0.11		5	4.03		−6.67 ± 0.09	211.8 ± 6.6	5	−7.57 ± 0.18	170.6 ± 20.2	7
Dopamine	−3.31	5					3.86		−6.34 ± 0.08	182.0 ± 13.1	6	−7.04 ± 0.13	151.3 ± 28.5	6
A61603	IC_50_ > 100 μM	5	−7.83 ± 0.05	−6.77 ± 0.05		11	>3.83		−6.66 ± 0.03	185.2 ± 9.3	5	−7.54 ± 0.12	160.7 ± 12.3	6
Oxymethazoline	−4.97	11	−8.77 ± 0.06	−7.74 ± 0.07		16	3.80		−7.78 ± 0.04	204.2 ± 16.2	5	#		
Octopamine	IC_50_ > 1 mM	5	−6.75 ± 0.10	−5.44 ± 0.08		5	>3.75		−5.32 ± 0.17	212.8 ± 14.2	5	−6.03 ± 0.18	175.8 ± 47.1	7
Isoprenaline	IC_50_ > 1 mM	5	−5.61 ± 0.16	−4.83 ± 0.10		5	>3.61		−4.78 ± 0.08	229.0 ± 9.5	5	−6.07 ± 0.16	156.5 ± 10.7	6
R‐phenylephrine	−3.96	5	−7.35 ± 0.13	−6.03 ± 0.14		6	3.39		−5.45 ± 0.04	203.2 ± 9.6	5	−6.78 ± 0.11	177.8 ± 19.9	6
Xylometazoline	−5.44	6	−8.75 ± 0.05	−7.75 ± 0.05		5	3.31		−7.47 ± 0.04	198.2 ± 10.5	5	#		
Para‐amino‐clonidine	−6.34	5	−9.64 ± 0.07	−7.95 ± 0.14		5	3.30		−7.45 ± 0.07	240.0 ± 10.7	5	−8.88 ± 0.13	177.4 ± 36.4	7
Medetomidine	−7.40	5	−10.62 ± 0.06	−9.69 ± 0.06		6	3.22		−9.35 ± 0.07	199.0 ± 19.4	5	−9.19 ± 0.22	195.3 ± 26.2	7
Dexmedetomidine	−7.66	6	−10.86 ± 0.06	−9.88 ± 0.06		7	3.20		−9.43 ± 0.05	171.3 ± 5.7	6	−9.24 ± 0.18	181.8 ± 61.2	7
Synephrine	−3.32	5	−6.47 ± 0.11	−5.22 ± 0.08		6	3.15		100 μM	186.2 ± 16.3	5	−6.12 ± 0.12	173.8 ± 28.5	6
BHT920	−5.77	5	−8.88 ± 0.09	−7.69 ± 0.08		6	3.11		−7.33 ± 0.07	217.0 ± 7.3	5	−8.34 ± 0.11	158.5 ± 24.6	6
ST‐91	−5.66	6	−8.77 ± 0.08	−7.52 ± 0.09		5	3.11		−7.20 ± 0.08	190.2 ± 8.7	5	−8.00 ± 0.16	195.2 ± 18.9	6
Guanfacine	−5.57	6	−8.66 ± 0.03	−7.61 ± 0.05		13	3.09		−7.47 ± 0.01	200.3 ± 24.4	6	−8.46 ± 0.17	177.2 ± 27.8	6
Detomidine	−7.15	5	−10.21 ± 0.04	−9.20 ± 0.05		5	3.06		−8.81 ± 0.06	176.0 ± 12.5	5	−8.98 ± 0.20	146.5 ± 20.7	7
Naphazoline	−5.80	5	−8.80 ± 0.05	−7.59 ± 0.07		12	3.00		−7.41 ± 0.08	227.6 ± 13.6	5	−8.30 ± 0.21	176.7 ± 25.9	7
Guanabenz	−6.02	5	−9.00 ± 0.08	−7.92 ± 0.06		19	2.98		−7.90 ± 0.05	194.9 ± 25.1	6	−8.54 ± 0.12	175.73 ± 28.9	6
Brimonidine	−5.47	5	−8.42 ± 0.06	−7.24 ± 0.05		16	2.95		−7.11 ± 0.05	222.4 ± 19.6	5	−7.78 ± 0.17	201.7 ± 18.4	8
BHT933	−4.46	5	−7.40 ± 0.12	−6.16 ± 0.09		6	2.94		−5.90 ± 0.10	217.0 ± 7.3	5	−7.07 ± 0.16	176.3 ± 19.9	6
Moxonidine	−4.58	5	−7.52 ± 0.04	−6.48 ± 0.04		8	2.94		−6.11 ± 0.06	238.0 ± 24.0	5	−7.14 ± 0.09	199.3 ± 21.0	7
UK14304	−5.55	5	−8.48 ± 0.08	−7.40 ± 0.04		5	2.93		−7.12 ± 0.06	237.8 ± 13.7	5	−8.30 ± 0.16	161.3 ± 20.7	6
Allyphenyline	−5.68	5	−8.50 ± 0.17	−6.93 ± 011		8	2.82		−6.68 ± 0.10	238.8 ± 19.9	5	−7.51 ± 0.10	182.1 ± 40.5	7
Methoxamine	−3.63	5	−6.40 ± 0.11	−5.13 ± 0.07		5	2.77		−4.61 ± 0.07	271.4 ± 28.5	5	−6.18 ± 0.08	151.8 ± 21.6	6
RWJ52353	IC_50_ > 10 μM	5	−7.68 ± 0.05	−6.53 ± 0.07		6	>2.68		−6.30 ± 0.02	237.8 ± 31.3	5	−7.19 ± 0.22	163.8 ± 20.7	6
Cirazoline	−5.17	5	−7.67 ± 0.07	−6.29 ± 0.07		9	2.50		−6.13 ± 0.06	221.0 ± 9.7	5	−6.91 ± 0.14	176.8 ± 20.8	6
Xylazine	−5.20	5	−7.65 ± 0.05	−6.41 ± 0.04		11	2.45		−6.26 ± 0.06	230.6 ± 21.5	5	−7.42 ± 0.12	206.6 ± 46.2	6
Etilefrine	−3.38	5	−5.83 ± 0.13	>100 μM		5	2.45		100 μM	194.4 ± 10.2	5	−5.63 ± 0.18	203.0 ± 61.2	6
Tetrahydrozoline	−5.25	6	−7.63 ± 0.07	−6.54 ± 0.06		5	2.38		−6.31 ± 0.10	217.6 ± 5.0	5	−7.44 ± 0.16	183.6 ± 26.8	6
Clonidine	−6.34	5	−8.63 ± 0.07	−7.42 ± 0.07		8	2.29		−7.24 ± 0.03	216.4 ± 6.4	5	−7.95 ± 0.14	217.2 ± 20.4	6
Amitraz	−5.29	5	−7.45 ± 0.09	−6.40 ± 0.09		5	2.16		−5.83 ± 0.11	190.2 ± 32.4	4	−6.53 ± 0.14	169.2 ± 16.1	6
Tizanidine	−5.78	5	−7.83 ± 0.12	−6.31 ± 0.11		6	2.05		−6.22 ± 0.07	168.3 ± 12.8	7	−7.01 ± 0.14	181.0 ± 47.2	6
Dihydroergotamine	−7.49	5	−9.53 ± 0.14	−8.49 ± 0.17		7	2.04		−8.13 ± 0.18	215.8 ± 7.0	6	#		
Dobutamine	−4.57	5	−6.53 ± 0.11	−5.94 ± 0.08		7	1.96		−5.43 ± 0.07	104.0 ± 6.2	5	−6.61 ± 0.15	177.0 ± 47.1	6
Bromocriptine	−6.90	5	−8.77 ± 0.17	−7.76 ± 0.18		7	1.87		−7.42 ± 0.23	131.5 ± 13.1	6	−8.67 ± 0.17^a^	166.6 ± 15.2	6
Rilmenidine	−5.40	5	−7.20 ± 0.10	−6.05 ± 0.07		5	1.80		−5.40 ± 0.12	241.2 ± 26.7	5	−7.12 ± 0.11	149.5 ± 27.4	7
T‐CG 1000	−6.01	5	−7.78 ± 0.08	−6.89 ± 0.05		5	1.77		−6.46 ± 0.05	115.6 ± 8.6	5	−7.40 ± 0.15	161.8 ± 10.5	7
Ephedrine	−3.84	5	−5.53 ± 0.11	−4.12 ± 0.22		5	1.69		1 mM	77.8 ± 8.1	5	−5.05 ± 0.11	145.8 ± 20.4	7
Atipamezole	−7.85	5	−9.39 ± 0.09	−8.06 ± 0.16		5	1.54		−7.67 ± 0.05	116.8 ± 15.6	5	−8.05 ± 0.20	168.8 ± 25.6	7
Buspirone	−4.62	5	−5.50 ± 0.16		36.1 ± 6.6	5	0.88		<10%		5	−5.32 ± 0.15	173.8 ± 24.1	5
Chloroethylclonidine	−4.35	5	No response			5			No response		5	−6.48 ± 0.11	137.7 ± 27.9	5
Salmeterol	−4.74	5	No response			5			No response		5			
Formoterol	IC_50_ > 100 μM	5	No response			5			No response		6			
Fenoterol	IC_50_ > 1 mM	5	No response			5			No response		6			
Salbutamol	IC_50_ > 1 mM3	5	No response			5			No response		5			
Midodrine	No binding	5	No response			5			100 μM	25.8 ± 5.8	5	100 μM	134.4 ± 17.4	8

*Note*: # these compounds stimulate ERK1/2‐phosphorylation in parent CHO cells^39^ so measurements were not made in this cell line.

^a^ bromocriptine also stimulated a response in parent CHO cells (see results, log EC_50_–6.93) but as this is far less potent that the response in CHO‐α2B cells (log EC_50_–8.67), it is included here as the CHO‐α2B response is likely to be α2B‐receptor mediated.

Most ligands had a biphasic CRE‐SPAP response in the CHO‐α2B cell line (Table 2, Supplementary Figures S3 and S4), likely due to its high expression of α2B‐adrenoceptors (13 102 fmoL/mg protein^40^). Affinity was also assessed, and compounds ranked in order of intrinsic efficacy (Table 2).

### 3.7 CHO‐α2C cells

**FIGURE 5:**
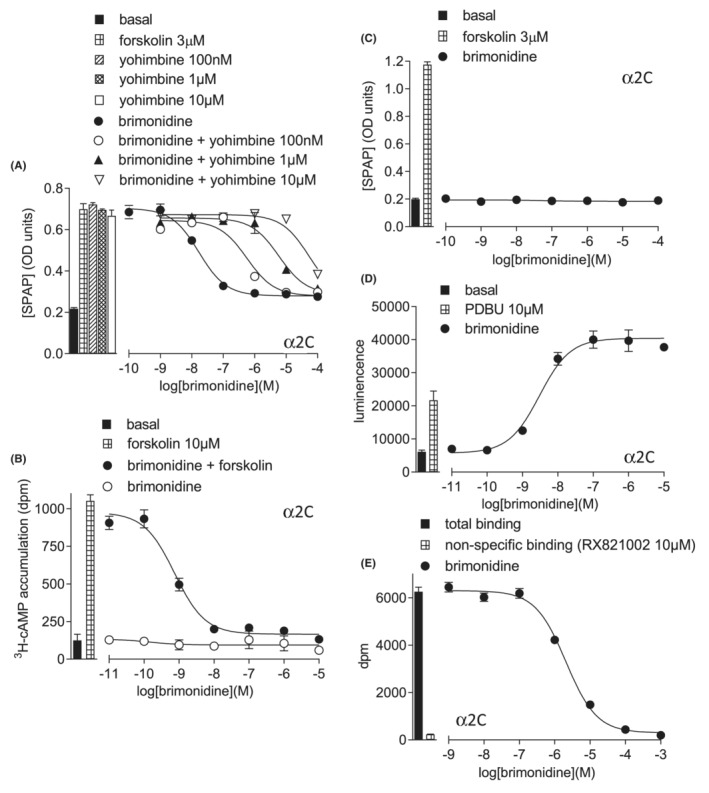
Responses to brimonidine in CHO‐α2C cells. (A) CRE‐SPAP production in the presence of 3 μM forskolin, in the presence and absence of yohimbine. Bars represent basal CRE‐SPAP production, that in response to 3 μM forskolin alone, and that in response to yohimbine 100 nM, 1 μM and 10 μM alone. (B) ^3^H‐cAMP accumulation in response to brimonidine in the absence and presence of 10 μM forskolin. Bars represent basal ^3^H‐cAMP accumulation and that in response to 10 μM forskolin. (C) CRE‐SPAP production in the absence of forskolin. Bars represent basal CRE‐SPAP production and that in response to 3 μM forskolin. (D) ERK1/2‐phosphorylation in response to brimonidine. Bars represent basal ERK1/2‐phosphorylation and that in response to 10 μM PDBu. and (E) inhibition of ^3^H‐rauwolscine binding in whole CHO‐α2B cells in response to brimonidine. Bars represent total binding and non‐specific binding as determined by 10 μM RX821002. The concentration of ^3^H‐rauwolscine in this experiment was 0.84 nM. Data points are mean ± SEM of triplicate determinations in all cases.

In the CHO‐α2C cells, brimonidine inhibited the forskolin‐stimulated CRE‐SPAP production in a manner best described by a monophasic sigmoidal response (log IC_50_ −8.00 ± 0.06, 82.9 ± 2.0% inhibition of 3 μM forskolin response, *n* = 17; Figure 5A, Table 3). In keeping with this, there was no stimulatory CRE‐SPAP response in the absence of forskolin (Figure 5C). The cAMP response was similar (log IC_50_ −8.96 ± 0.14, 97.7 ± 4.8% inhibition of 10 μM forskolin, *n* = 6, Figure 5B), with no response seen in the absence of forskolin (*n* = 6). Once again, the ERK1/2‐phosphorylation response (log EC_50_–8.21 ± 0.23, *n* = 8, Figure 5D) occurred at a similar potency to the inhibitory responses, as it was for all agonists (Supplementary Figures S5 and S6, Table 3). Affinity was obtained and ligands were once again ranked in order of efficacy ratio (Table 3).

**TABLE 3:** Data obtained in CHO‐α2C cells. Log K_D_ values from ^3^H‐rauwolscine whole cell binding (see Table 4 for mean ± SEM and n numbers); inhibition log IC_50_ and % inhibition from the 3 μM forskolin control; log efficacy ratio (K_D_/IC_50_); log EC_50_ and % maximum response compared to 3 μM forskolin from CRE‐SPAP production in the absence of forskolin; and log EC_50_ and % maximum response compared to 10 μM PDBU from ERK1/2‐phosphorylation. The ligands are arranged in order of α2C intrinsic efficacy ratio (K_D_/IC_50_)

CHO‐α2C	binding		CRE‐SPAP (with forskolin)					CRE‐SPAP (without forskolin)			ERK1/2‐phosphorylation		
	Log K_D_	*n*	Log IC_50_ (Gi)	Log EC_50_ (Gs)	% inhibition	*n*	Log efficacy ratio	Log EC_50_ (Gs)	% 3 μM forskolin	*n*	Log EC_50_	% 10 μM PDBu	*n*
A61603	IC_50_ > −4	5	−6.68 ± 0.09		80.5 ± 3.0	12	>2.68	No response		5	−7.26 ± 0.15	149.2 ± 30.3	6
UK14304	−6.08	5	−8.19 ± 0.08		80.3 ± 1.8	5	2.11	No response		5	−8.87 ± 0.26	144.1 ± 20.8	7
Isoprenaline	IC_50_ > −3	5	−5.04 ± 0.10		95.0 ± 5.2	5	>2.04	No response		5	−6.06 ± 0.18	130.7 ± 11.1	6
Brimonidine	−5.97	5	−8.00 ± 0.06		82.9 ± 2.0	17	2.03	No response		9	−8.21 ± 0.23	160.9 ± 11.5	8
Noradrenaline	−4.49	9	−6.51 ± 0.10		83.0 ± 3.1	10	2.02	No response		6	−7.72 ± 0.16	142.3 ± 10.7	5
Adrenaline	−4.88	10	−6.74 ± 0.17		80.5 ± 2.5	8	1.86	No response		11	−7.55 ± 0.12	140.0 ± 9.0	7
Medetomidine	−7.49	5	−9.29 ± 0.04		86.4 ± 2.1	6	1.80	No response		6	−9.71 ± 0.18	159.3 ± 16.4	7
RWJ52353	−4.67	5	−6.47 ± 0.10		76.5 ± 5.6	5	1.80	No response		5	−7.74 ± 0.09	143.5 ± 14.0	5
Para‐amino‐clonidine	−6.31	5	−8.09 ± 0.07		82.3 ± 2.2	8	1.78	No response		8	−8.68 ± 0.13	132.6 ± 16.1	7
Dopamine	−3.89	5	−5.62 ± 0.09		87.0 ± 3.7	9	1.73	No response		6	−6.95 ± 0.13	120.8 ± 13.2	6
Dexmedetonidine	−7.52	6	−9.16 ± 0.09		73.4 ± 4.4	12	1.64	No response		12	−9.58 ± 0.13	153.0 ± 18.5	7
R‐phenylephrine	−4.59	5	−6.20 ± 0.10		77.5 ± 3.4	6	1.61	No response		5	−6.57 ± 0.15	157.0 ± 15.4	6
Moxonidine	−4.75	5	−6.32 ± 0.06		76.4 ± 5.3	8	1.57	No response		5	−6.93 ± 0.13	168.8 ± 16.1	7
Metaraminol	−4.54	5	−6.10 ± 0.04		85.5 ± 1.8	5	1.56	No response		5	−6.80 ± 0.15	158.8 ± 22.2	7
Xylazine	−5.22	5	−6.75 ± 0.04		81.8 ± 3.6	9	1.53	No response		5	−7.14 ± 0.16	168.2 ± 22.8	6
BHT920	−5.99	5	−7.41 ± 0.10		86.8 ± 6.0	6	1.42	No response		5	−8.18 ± 0.12	144.8 ± 23.2	6
Detomidine	−7.11	5	−8.48 ± 0.12		80.8 ± 4.5	10	1.37	No response		6	−9.11 ± 0.16	140.1 ± 12.9	7
α‐methylnorepin ephrine	−5.16	5	−6.52 ± 0.12		85.2 ± 3.0	13	1.36	No response		8	−7.72 ± 0.14	167.0 ± 22.9	7
Guanfacine	−5.92	6	−7.24 ± 0.12		78.5 ± 5.0	10	1.32	No response		5	−7.98 ± 0.16	152.4 ± 24.3	7
Octopamine	IC_50_ ~ −3	5	−4.28 ± 0.15		86.3 ± 5.2	5	>1.28	No response		5	−5.11 ± 0.17	153.8 ± 17.9	7
ST‐91	−6.18	6	−7.46 ± 0.09		76.5 ± 4.4	5	1.28	No response		5	−7.93 ± 0.17	154.0 ± 15.0	6
Guanabenz	−6.35	5	−7.49 ± 0.08		69.8 ± 4.5	17	1.14	No response		5	−8.38 ± 0.12	159.6 ± 9.0	6
Etilefrine	−3.91	5	−4.92 ± 0.08		86.2 ± 7.2	5	1.01	No response		5	−5.50 ± 0.17	182.2 ± 9.6	6
Methoxamine	−3.95	5	−4.96 ± 0.17		72.3 ± 13.6	6	1.01	No response		6	−5.63 ± 0.10	131.0 ± 19.2	6
Oxymethazoline	−6.42	10	−7.38 ± 0.9		71.6 ± 4.4	11	0.96	No response		5	#		
Clonidine	−6.56	5	−7.46 ± 0.07		78.5 ± 2.4	7	0.90	No response		6	−7.84 ± 0.20	165.8 ± 7.3	6
Amitraz	−5.69	5	−6.45 ± 0.09		89.4 ± 2.4	5	0.76	No response		5	−6.84 ± 0.24	140.0 ± 19.9	6
Naphazoline	−6.40	5	−7.12 ± 0.07		81.3 ± 4.2	9	0.72	No response		5	−7.90 ± 0.17	157.8 ± 12.6	6
Tizanidine	−5.83	5	−6.52 ± 0.07		72.1 ± 4.4	5	0.69	No response		5	−6.82 ± 0.16	133.6 ± 17.9	6
BHT933	−5.32	5	−5.94 ± 0.08		86.0 ± 4.6	6	0.62	No response		5	−6.63 ± 0.18	150.0 ± 19.7	6
Synephrine	−3.92	5	−4.46 ± 0.14		88.1 ± 8.5	5	0.54	No response		5	−5.49 ± 0.18	112.8 ± 10.7	6
Allyphenyline	−6.67	5	−7.20 ± 0.10		66.8 ± 4.7	5	0.53	No response		5	−7.93 ± 0.19	134.5 ± 17.9	6
Rilmenidine	−5.81	5	−6.33 ± 0.08		69.0 ± 7.9	5	0.52	No response		5	−7.31 ± 0.17	125.2 ± 15.6	6
chloroethylclonidine	−5.49	5	−5.99 ± 0.06		84.6 ± 4.9	6	0.50	No response		5	−6.21 ± 0.17	103.0 ± 17.2	8
Cirazoline	−6.07	5	−6.48 ± 0.08		74.2 ± 2.1	8	0.41	No response		5	−7.08 ± 0.23	139.1 ± 10.2	6
Tetrahydrozoline	−6.07	6	−6.47 ± 0.07		60.7 ± 3.2	5	0.40	No response		5	−6.90 ± 0.15	139.6 ± 19.8	6
Xylometazoline	−6.97	6	−7.20 ± 0.10		57.2 ± 3.9	5	0.23	No response		5	#		
Dihydroergotamine	−9.45	5	−9.27 ± 0.21		23.8 ± 5.7	5	−0.18	No response		5	#		
Bromocriptine	−7.63	5	−7.43 ± 0.10		62.3 ± 10.0	5	−0.20	No response		5	−8.34 ± 0.17^a^	126.0 ± 28.9	6
Dobutamine	−5.26	5	−4.68 ± 0.07		83.0 ± 9.1	5	−0.58	No response		5	−6.12 ± 0.15	125.5 ± 18.0	7
T‐CG 1000	−6.75	5	−6.12 ± 0.05		50.2 ± 5.1	5	−0.63	No response		5	−7.05 ± 0.14	87.5 ± 14.2	7
Atipamezole	−8.48	5	−6.70 ± 0.16		15.8 ± 7.5	7	−1.78	No response		5	−8.08 ± 0.11	63.7 ± 8.3	7
Midodrine	IC_50_ > 1 mM	5	IC_50_ > 100 μM			5		No response		5	100 μM	80.9 ± 15.6	8
Buspirone	−6.15	5	No response			5		No response		5	−5.70 ± 0.16	25.2 ± 7.7	6
Ephedrine	−4.40	5	No response			5		No response		5	−4.48 ± 0.13	88.0 ± 12.5	7
Salmeterol	−5.28	5	No response			5		No response		5			
Fenoterol	−3.82	5	No response			5		No response		6			
Formoterol	IC_50_ > 100 μM	5	No response			5		No response		6			
Salbutamol	IC_50_ ~ −3	5	No response			5		No response		5			

*Note*: # these compounds stimulate ERK1/2‐phosphorylation in parent CHO cells^39^ so measurements were not made in this cell line.

^a^ bromocriptine also stimulated a response in parent CHO cells (see results, log EC_50_–6.93) but as this is far less potent that the response in CHO‐α2C cells (log EC_50_–8.34), it is included here as the CHO‐α2C response is likely to be α2C‐receptor mediated.

**TABLE 4:** Log K_D_ values obtained from inhibition of ^3^H‐rauwolscine binding to the human α2A, α2B and α2C‐adrenoceptors in living cells. Values represent mean ± SEM of n separate experiments. Selectivity ratios are also given where a ratio of 1 demonstrates no selectivity for a given receptor subtype over another. Thus oxymetazoline has 200‐fold higher affinity for the α2A than the α2B‐adrenoceptor. Compounds are arranged in order of α2A‐selectivity.

	Log *K*_*D*_ values determined from ^3^H‐rauwolscine whole cell binding						Selectivity ratios					
	CHO‐α2A	*n*	CHO‐α2B	*n*	CHO‐α2C	*n*	α2A vs α2B		α2A vs α2C		α2B vs α2C	
Oxymethazoline	−7.27 ± 0.03	11	−4.97 ± 0.04	11	−6.42 ± 0.07	10	200		7.1			28.2
Xylometazoline	−7.62 ± 0.04	6	−5.44 ± 0.09	6	−6.97 ± 0.04	6	151		4.5			33.9
Bromocryptine	−8.25 ± 0.04	5	−6.90 ± 0.01	5	−7.63 ± 0.05	5	22.4		4.2			5.4
Tetrahydrozoline	−6.49 ± 0.05	6	−5.25 ± 0.05	6	−6.07 ± 0.03	6	17.4		2.6			6.6
Allyphenyline	−6.92 ± 0.03	5	−5.68 ± 0.05	5	−6.67 ± 0.08	5	17.4		1.8			9.8
Naphazoline	−7.01 ± 0.06	5	−5.80 ± 0.04	5	−6.40 ± 0.04	5	16.2		4.1			4.0
Cirazoline	−6.38 ± 0.05	5	−5.17 ± 0.04	5	−6.07 ± 0.10	5	16.2		2.0			7.9
Chloroethylclonidine	−5.47 ± 0.03	5	−4.35 ± 0.04	5	−5.49 ± 0.07	5	13.2		1.0			13.8
Dihydroergotamine	−8.59 ± 0.02	5	−7.49 ± 0.03	5	−9.45 ± 0.11	5	12.6			7.2		91.2
T‐CG 1000	−7.08 ± 0.03	5	−6.01 ± 0.03	5	−6.75 ± 0.09	5	11.7		2.1			5.5
Guanfacine	−6.58 ± 0.04	6	−5.57 ± 0.02	6	−5.92 ± 0.06	6	10.2		4.6			2.2
Guanabenz	−6.96 ± 0.01	6	−6.02 ± 0.05	5	−6.35 ± 0.05	5	8.7		4.1			2.1
R‐phenylephrine	−4.89 ± 0.03	5	−3.96 ± 0.03	5	−4.59 ± 0.07	5	8.5		2.0			4.3
Brimonidine	−6.37 ± 0.07	5	−5.47 ± 0.08	5	−5.97 ± 0.02	5	7.9		2.5			3.2
UK14304	−6.41 ± 0.01	5	−5.55 ± 0.05	5	−6.08 ± 0.06	5	7.2		2.1			3.4
Amitraz	−6.13 ± 0.04	5	−5.29 ± 0.07	5	−5.69 ± 0.03	5	6.9		2.8			2.5
Synephrine	−4.05 ± 0.01	5	−3.32 ± 0.02^app^	5	−3.92 ± 0.05^app^	5	5.4		1.3			4.0
Atipamezole	−8.50 ± 0.08	5	−7.85 ± 0.04	5	−8.48 ± 0.09	5	4.5		1.0			4.3
Buspirone	−5.24 ± 0.02	5	−4.62 ± 0.06	5	−6.15 ± 0.03	5	4.2			8.1		33.9
Ephedrine	−4.46 ± 0.04	5	−3.84 ± 0.07^app^	5	−4.40 ± 0.10	5	4.2		1.1			3.6
ST‐91	−6.15 ± 0.02	6	−5.66 ± 0.04	6	−6.18 ± 0.09	6	3.1			1.1		3.3
Moxonidine	−5.02 ± 0.02	5	−4.58 ± 0.04	5	−4.75 ± 0.04	5	2.8		1.9			1.5
BHT933	−4.89 ± 0.04	5	−4.46 ± 0.07	5	−5.32 ± 0.05	5	2.7			2.7		7.2
Rilmenidine	−5.81 ± 0.04	5	−5.40 ± 0.06	5	−5.81 ± 0.09	5	2.6		1.0			2.6
Methoxamine	−4.03 ± 0.03^app^	5	−3.63 ± 0.08^app^	5	−3.95 ± 0.12^app^	5	2.5		1.2			2.1
Clonidine	−6.72 ± 0.03	5	−6.34 ± 0.06	5	−6.56 ± 0.07	5	2.4		1.4			1.7
Etilefrine	−3.71 ± 0.06^app^	5	−3.38 ± 0.04^app^	5	−3.91 ± 0.02^app^	5	2.1			1.6		3.4
Detomidine	−7.41 ± 0.04	5	−7.15 ± 0.06	5	−7.11 ± 0.06	5	1.8		2.0		1.1	
Tizanidine	−5.97 ± 0.06	5	−5.78 ± 0.07	5	−5.83 ± 0.08	5	1.5		1.4			1.1
BHT920	−5.94 ± 0.04	5	−5.77 ± 0.05	5	−5.99 ± 0.03	5	1.5			1.1		1.7
Metaraminol	−4.28 ± 0.03	5	−4.11 ± 0.05^app^	8	−4.54 ± 0.06	5	1.5			1.8		2.7
Adrenaline	−3.74 ± 0.09	10	−3.56 ± 0.11	9	−4.88 ± 0.11	10	1.5			13.8		20.9
Medetomidine	−7.52 ± 0.06	5	−7.40 ± 0.01	5	−7.49 ± 0.05	5	1.3		1.1			1.2
Dobutamine	−4.69 ± 0.01	5	−4.57 ± 0.05	5	−5.26 ± 0.04	5	1.3			3.7		4.9
Dopamine	−3.39 ± 0.04	5	−3.31 ± 0.08	5	−3.89 ± 0.02	5	1.2			3.2		3.8
Dexmedetonidine	−7.70 ± 0.04	6	−7.66 ± 0.03	6	−7.52 ± 0.06	6	1.1		1.5		1.4	
Noradrenaline	−3.57 ± 0.03	9	−3.52 ± 0.11	9	−4.49 ± 0.07	9	1.1			8.3		9.3
Para‐amino‐clonidine	−6.35 ± 0.03	5	−6.34 ± 0.04	5	−6.31 ± 0.04	5	1.0		1.1		1.1	
α‐methylnorepin ephrine	−3.69 ± 0.04^app^	5	−3.80 ± 0.11^app^	5	−5.16 ± 0.03	5		1.3		29.5		22.9
Salmeterol	−4.76 ± 0.05^app^	5	−4.74 ± 0.08^app^	5	−5.28 ± 0.07	5	1.0			3.3		3.5
Xylazine	−4.94 ± 0.09	5	−5.20 ± 0.05	5	−5.22 ± 0.02	5		1.8		1.9		1.0
RWJ52353	−4.76 ± 0.08^app^	5	IC_50_ > 10 μM	5	−4.67 ± 0.08^app^	5			1.2			
Fenoterol	−3.46 ± 0.05^app^	5	IC_50_ > 1 mM	5	−3.82 ± 0.03^app^	5				2.3		
Octopamine	−3.38 ± 0.03^app^	5	IC_50_ > 1 mM	5	IC_50_ ~ 1 mM	5						
A61603	IC_50_ ~ 100 μM	5	IC_50_ > 100 μM	5	IC_50_ > 100 μM	5						
Formoterol	IC_50_ > 100 μM	5	IC_50_ > 100 μM	5	IC_50_ > 100 μM	5						
Isoprenaline	IC_50_ > 1 mM	5	IC_50_ > 1 mM	5	IC_50_ > 1 mM	5						
Salbutamol	IC_50_ > 1 mM	5	IC_50_ > 1 mM	5	IC_50_ ~ 1 mM	5						
Midodrine	IC_50_ > 1 mM	5	No binding	5	IC_50_ > 1 mM	5						

^app^ the maximum concentration of competing ligand inhibited most but not all specific binding. An IC_50_ was determined by extrapolating the curve assuming that all specific binding would be inhibited if a higher concentration of competing ligand were possible. Thus an apparent *K*_*D*_ was calculated.

### 3.8 CHO‐β1 and CHO‐β2 cells

As expected the β‐AR agonists (e.g., fenoterol, formoterol and salbutamol) stimulated potent responses in the CHO‐β1 and CHO‐β2 cells, however significant agonist responses and measureable affinity were also seen in response to a few α‐agonists e.g., etilefrine, metaraminol, phenylephrine and methoxamine (Supplementary Figure S7, Table S1 binding affinity) and Table 2 CRE‐SPAP responses). There was no binding or CRE‐SPAP responses to any of the classical α2‐agonists e.g., brimonidine, clonidine, dexmedetomidine etc.

### 3.9 CHO‐CRE‐SPAP cells

There were no CRE‐SPAP responses to any of the agonist ligands examined the parental CHO‐CRE‐SPAP cell line (i.e. cells stably expressing the CRE‐SPAP reporter, but with no transfected receptor), either in the presence (looking for Gi responses) or absence (looking for Gs responses) of forskolin (Supplementary Table S2). Oxymetazoline, xylometazoline and dihydroergotamine have previously been demonstrated to stimulate ERK1/2‐phosphorylation agonist responses via a non‐α‐mediated mechanism in the parent cells (see^39^ for details). There were no other ERK1/2‐phosphorylation agonist responses in these cells with the exception of bromocriptine (log EC_50_ −6.93 ± 0.18, 21.4 ± 6.8% 10 μM PDBU), whose responses were considerably less potent and much smaller in amplitude than those seen in the α2A cell lines. The bromocriptine responses in Tables 1, 2, 3 are therefore highly likely to be occurring via the transfected α2‐adrenoceptors.

Of note, some Gi‐coupled receptors have been found to stimulate calcium responses (e.g., muscarinic M2 receptor^49^). Calcium/Gq‐coupling was not assessed as part of this study.

## 4 DISCUSSION

Certain α2‐agonists stimulate biphasic cAMP responses at α2‐adrenoceptors, with Gi‐cAMP inhibition at low concentrations followed by Gs‐mediated stimulation at higher concentrations. However, other ligands, of equal Gi‐mediated potency do not stimulate Gs. This study aimed to investigate this.

Brimonidine stimulated biphasic α2A‐adrenoceptor responses for both CRE‐SPAP production and ^3^H‐cAMP accumulation as previously observed.^17^, ^26^, ^27^, ^28^, ^29^, ^30^, ^31^, ^32^, ^47^ This Gi and Gs‐protein coupling is through third intracellular loop residues,^31^ and is similar to adenosine A1 receptor agonist responses.^41^ However, whilst moxonidine and naphazoline have similar Gi‐potency, only moxonidine stimulated a Gs‐response. This is similar to^33^'s observation that agonists with similar Gi‐responses (including full agonists) had different Gs‐responses. When extended to other α2‐agonists, a graded spectrum was seen from agonists with large Gs‐stimulatory components, through to those with none.

**FIGURE 6:**
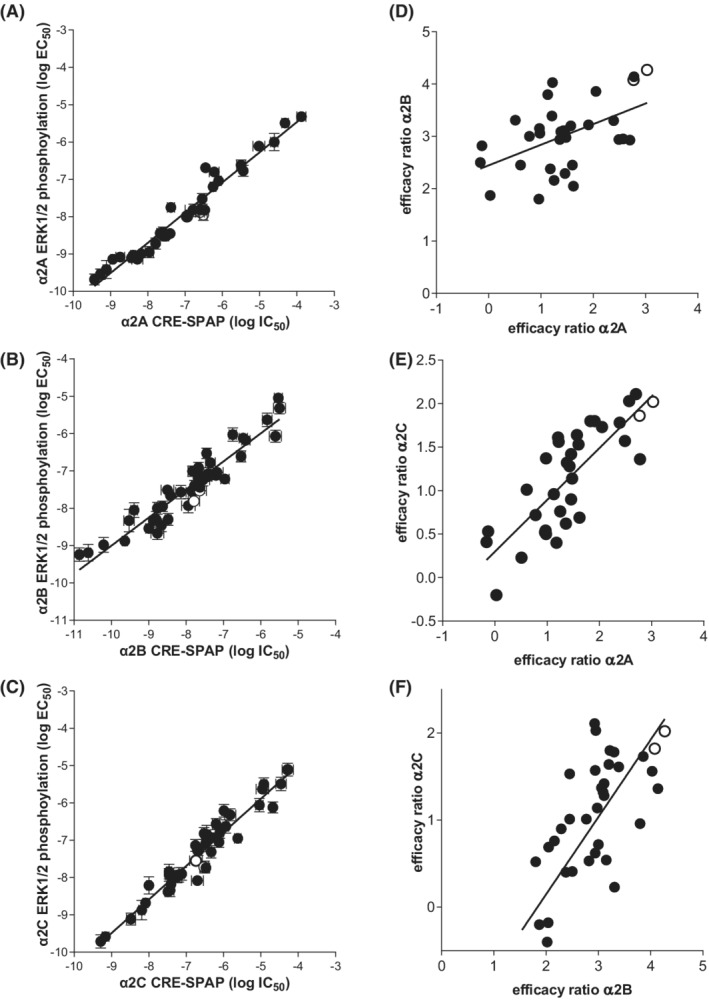
(A–C) Correlation plots of log IC_50_ determined from CRE‐SPAP production with the EC_50_ determined from ERK1/2‐phosphorylation in a) CHO‐α2A cells, (B) CHO‐α2B cells and (C) CHO α2C cells. Data point are mean ± SEM taken from Tables 1, 2, 3. The endogenous hormones adrenaline and noradrenaline are represented by open circles. The line is that of best fit. (D–F) Correlation plots of efficacy ratio (K_D_/IC_50_) for (D) α2A vs α2B, (E) α2A vs α2C and (F) α2B vs α2C as determined from whole cell binding affinity measurements and inhibition of forskolin‐stimulated CRE‐SPAP production. The endogenous hormones adrenaline and noradrenaline are represented by open circles. The line is that of best fit and the slope is not 1 and does not necessarily go through the origin as this represents a function of efficacy (i.e. differences in cell line which include receptor number, receptor‐effector coupling etc.). The data for oxymetazoline, xylometazoline and dihydroergotamine are not included in these plots as the compounds generated agonist ERK1/2‐phosphorylation responses in non‐transfected cells and are therefore non‐α2‐mediated responses. Compounds with the greatest perpendicular distance from the line represent compounds with the greatest degree of selective intrinsic efficacy.

As CRE‐SPAP responses can involve ERK1/2‐phosphorylation separately from the Gs‐cAMP pathway (biased signaling at β2‐adrenoceptor^50^), and previous reports of α2‐adrenoceptor ERK1/2‐phosphorylation,^51^, ^52^, ^53^ this was studied. Agonists stimulated ERK1/2‐phosphorylation with potencies (EC_50_ values) closely mirroring the Gi‐inhibitory response. Correlation plots of IC_50_ (Gi‐mediated 5 h CRE‐SPAP inhibition) vs EC_50_ (2–4 min ERK1/2‐phosphorylation) give straight lines (Figure 6A‐C). This agrees with others' observations that α2A‐ERK1/2‐phosphorylation is a Gi‐mediated response. Indeed PTX‐pre‐treatment abolished α2A‐ERK1/2‐phosphorylation responses.^51^, ^52^, ^53^ Thus ERK1/2‐phosphorylation biased signaling does not explain why only some agonists stimulate CRE‐SPAP production.

Studies with different receptor expression levels give hints. Of three α2A‐adrenoceptor cell lines studied, the higher the receptor expression level, the larger the Gs‐stimulation, including no Gs‐responses in the cell line with very low receptor expression. Others^27^, ^54^ report similar findings. So the ability to induce Gs‐responses depends upon the receptor reserve and ligands with biphasic responses appear monophasic in systems with low receptor reserve.

Ligand affinity was examined to enable the two properties of agonist ligands (affinity and intrinsic efficacy) to be studied separately and a measure of intrinsic efficacy (efficacy ratio) obtained. For brimonidine and moxonidine, the efficacy ratio was high (log 2.57 and 2.48 respectively), suggesting few receptors need occupying to stimulate agonist responses (i.e. the compounds had high intrinsic efficacy). Naphazoline had a lower efficacy ratio at 0.78 (lower intrinsic efficacy). Table 1, arranged in efficacy ratio order, shows that compounds with the highest intrinsic efficacy stimulated Gs‐responses, irrespective of their potency or affinity. Thus, high intrinsic efficacy enables some compounds to stimulate Gs‐responses.

This explains others' findings Eason et al.,^33^ reported that despite similar Gi‐inhibition, adrenaline, noradrenaline and brimonidine stimulated Gs‐responses whereas BHT920 and BHT933 did not. BHT933 and BHT920 are lower efficacy compounds (Table 1). Qu et al^47^ reported that a TM6 mutation (Y394N) reduced Gi‐potency by 1000‐10 000‐fold. The Gs‐response was also attenuated – likely due to loss of agonist affinity and/or intrinsic efficacy. Gs‐responses were exaggerated in a constitutively active α2A‐mutant with Gs‐responses left‐shifted compared to wild‐type and obliterating the Gi‐coupled response.^32^

Thus (1) high receptor reserve and (2) high ligand intrinsic efficacy are both required for observation of Gs‐coupling. What remains unknown, is how higher ligand concentrations induce a different conformational state that alters receptor‐G‐protein coupling, nor whether this phenomenon is relevant in native tissues or clinical responses. Interestingly, dexmedetomidine exhibits a biphasic blood pressure response in people, with low dose infusions reducing blood pressure and high dose infusions increasing blood pressure.^55^ This has been attributed to a loss of dexmedetomidine selectivity at higher doses,^16^ however it is tempting to consider it may, in part, be due to α2‐Gs‐activation. α2‐agonists used systemically in clinical practice (e.g., clonidine for hypertension, dexmedetomidine for sedation, guanfacine for ADHD, tizanidine for spasticity) are mid‐range, partial agonists.

The α2B‐adrenoceptor cell line has very high receptor expression, with biphasic responses and substantial Gs‐stimulation with many agonists. ERK1/2‐phosphorylation mirrored the Gi‐inhibitory CRE‐SPAP component (Figure 4) and the degree of Gs‐stimulatory response was again related to the intrinsic efficacy of the agonist compound.

The α2C‐adrenoceptor cell line had a lower receptor expression and although agonists inhibited both CRE‐SPAP and cAMP responses (Gi), no Gs‐responses were seen (similar to low expressing α2A cell line [cell line 3] Supplementary Figure S1). Once again, the ERK1/2‐phosphorylation mirrored the Gi‐inhibition (Figure 5). This cell line appears to have too little receptor reserve to observe Gs‐coupling. Kribben et al^53^ examined noradrenaline and octopamine responses in CHO cells with similar α2A, α2B and α2C‐adrenoceptor receptor expression and found different degrees of Gs stimulation (α2B having the largest Gs‐responses). Thus different α2‐subtypes may also have different G‐protein coupling efficiencies.

As affinity and intrinsic efficacy measurements were made in all α2‐adrenoceptor subtypes under identical conditions, ligand affinity and rank orders of intrinsic efficacy can be directly compared. Furthermore, as identical conditions were used for α1‐adrenceptor measurements,^39^ comparison across all human α‐ and β1 and β2‐adrenoceptors is possible.

Oxymetazoline was the most affinity‐selective α2‐agonist (α2A affinity 200‐fold higher than α2B and 28‐fold higher than α2C‐adrenoceptors) similar to comparisons from human colonic adenocarcinoma cells (α2A), neonatal rat lung (α2B) and opossum kidney cells (α2C)^23^, ^24^ and in rat,^25^ guinea pig^28^ and pig.^56^ Other similarities exist ‐ guanfacine and guanabenz had 10‐fold higher α2A than α2B affinity similar to.^25^ Although precise values vary, not least because of species differences, the pattern of higher affinity for dexmedetomidine and medetomidine, followed by clonidine and guanabenz and lower affinity for catecholamines and xylazine is common across studies.^17^, ^25^, ^28^, ^57^, ^58^, ^59^ However, there was little α2‐selective affinity for the other α‐agonists, also noted by^17^ and no α2B‐selective agonists.

Oxymetazoline (α2A log *K*_*D*_ −7.27), and related xylometazoline, also have high α1A‐adrenoceptor affinity (α1A log *K*_*D*_ −7.19^39^) but not for α1B/D, α2B/C or β1/2‐adrenoceptors. These compounds have selectivity across receptor subtypes, rather than between subtypes. They also activate non‐adrenoceptor responses (including the ERK1/2‐phosphorylation in these cells, probably via native CHO 5HT‐1B receptors^60^).

As expected, catecholamines had high intrinsic efficacy. Medetomidine, and stereoisomer dexmedetomidine, were the most potent agonists for all α2‐subtypes, but also had the highest affinities (as in^28^). Thus, the intrinsic efficacy of these is only mid‐range. This high potency has been reported before.^17^'s conclusion that dexmedetomidine was their most potent α2‐agonist compound, more than catecholamines, is absolutely correct but only part of the story. Dexmedetomidine did not have the highest intrinsic efficacy (i.e. not the most efficacious agonist) either in terms of maximum response or if efficacy ratios are calculated using their data (again mid‐ranking). As higher intrinsic efficacy determines the Gs‐coupling, this explains why, despite being the most potent agonists, medetomidine and dexmedetomidine did not elicit the largest Gs‐stimulation.

There is some correlation between the intrinsic efficacy of compounds at the different α2‐subtypes with some agonists being more efficacious at all three subtypes (e.g., catecholamines) and others having lower efficacy (e.g., clonidine and rilmenidine). However, there are some differences (Figure 6D‐F). Brimonidine/UK14304 are highly efficacious α2A and α2C‐agonists (both present in brain), with medetomidine and dexmedetomidine being less efficacious. However, the rank order of compounds is reversed at α2B‐adrenoceptors with medetomidine and dexmedetomidine being more efficacious than brimonidine/UK14304. This rank order is different for other compounds – oxymetazoline and xylometazoline are higher up the rank order in α2B and lower in α2A and α2C‐subtypes. This suggests there may be some subtype selectivity for intrinsic efficacy.

A61603 was a very efficacious ligand at all α‐adrenoceptors (although not β1/β2‐adrenoceptors). However, it has 1000‐fold higher α1A‐affinity than for any other α‐adrenoceptor, giving rise to more potent α1A functional responses. A61603 is an affinity‐selective α1A‐agonist. Interestingly at α2A‐adrenoceptors, A61603 was the only compound where the Gs‐response was lower than predicted from Gi‐potency and intrinsic efficacy. The reason is unknown, although the binding was so poor that affinity (and efficacy ratio) could not be accurately established.

Perhaps more interesting is the comparison between α1 and α2‐subtypes. Dexmedetomidine has 100‐fold higher affinity for α2 than α1‐adrenoceptor subtypes with mid‐range efficacy at all six α‐subtypes, suggesting that affinity is largely driving the higher α2 vs α1‐potency of dexmedetomidine responses. However, brimonidine only has a 10‐fold higher α2 than α1‐affinity but very high α2‐intrinsic efficacy (giving potent responses) and low α1 intrinsic efficacy. The α2‐selectivity of brimonidine appears to be driven more by α2‐selective intrinsic efficacy with less reliance on selective affinity.

There are examples of the reverse. R‐phenylephrine, etilefrine, metaraminol and methoxamine have similar affinity across all α‐subtypes but are highly efficacious at α1‐adrenoceptors with low efficacy at α2A and α2C‐subtypes (interestingly α2B is once again a little different). These compounds α1‐selective functional responses are being driven by α1‐selective intrinsic activity, whilst A61603, above, has α1A‐selective affinity.

In conclusion, both (1) system high receptor reserve and (2) agonist high intrinsic efficacy are required for α2‐Gs‐mediated responses to be observed. From the Gi‐mediated efficacy ratio (binding *K*_*D*_/Gi‐IC_50_), the degree of Gs‐stimulation observed within a given system can be predicted. It remains to be determined whether this Gs‐coupling is clinically relevant and the precise receptor conformational changes that occur, given the structural diversity of compounds with high intrinsic efficacy.

This study also shows the importance of separating affinity and intrinsic efficacy to understand agonist ligand responses. Some α‐ligands are selective because of affinity (A61603:α1A and dexmedetomidine:α2) whilst others are selective due to intrinsic efficacy (methoxamine/etilefrine:α1 and brimonidine:α2). A detailed knowledge of these agonist characteristics is vital for improving computer‐based drug design.^61^

## AUTHOR CONTRIBUTIONS

JGB designed the research study. RGWP, JA and JGB performed the research. JGB and JA analyzed the data. JGB wrote the paper.

## CONFLICT OF INTEREST

JGB has been on the Scientific Advisory Board for CuraSen Therapeutics since 2019.

## ETHICAL STATEMENT

No animals, human tissue, human volunteers or patients were used in this study.

## Supporting information


Data S1

## Data Availability

Further information and requests for data and reagents should be directed to and will be fulfilled by the corresponding author, Jillian Baker. Please contact jillian.baker@nottingham.ac.uk
