# Supplementary material for: The signaling and selectivity of α‐adrenoceptor agonists for the human α2A, α2B and α2C‐adrenoceptors and comparison with human α1 and β‐adrenoceptors
Source: Pharmacol Res Perspect. 2022 Sep 13;10(5):e01003. doi: 10.1002/prp2.1003 (PMC9471048; doi:10.1002/prp2.1003)
Supplement: Supplementary file 1 — Data S1 [file PRP2-10-e01003-s001.docx]

The signalling and selectivity of α-adrenoceptor agonists for the human α2A, α2B and α2C-adrenoceptors and comparison with human α1 and β-adrenoceptors.

## Richard G.W. Proudman, Juliana Akinaga, Jillian G. Baker

Cell Signalling Research Group,

Division of Physiology, Pharmacology and Neuroscience,

School of Life Sciences,

C Floor Medical School,

Queen’s Medical Centre,

University of Nottingham,

Nottingham,

NG7 2UH,

UK.

Supplementary figure 1 responses in CHO-α2A lines of different expression levels

Supplementary figure 2 dexmedetomidine responses in CHO-α2A cells

Supplementary figure 3 moxonidine responses in CHO-α2B cells

Supplementary figure 4 naphazoline responses in CHO-α2B cells

Supplementary figure 5 moxonidine responses in CHO-α2C cells

Supplementary figure 6 naphazoline responses in CHO-α2C cells

Supplementary figure 7 etilefrine responses in CHO-β1 and CHO-β2 cells

Supplementary table 1 ligand sources, binding K_D_ values obtained in CHO-β1 and CHO-β2 cells and maximum concentrations used in binding studies

Supplementary table 2 CRE-SPAP production in CHO-β1, CHO-β2 and CRE-SPAP cells and maximum concentrations used in funstional studies

Supplementary Figure 1 - responses in CHO-α2A lines of different expression levels


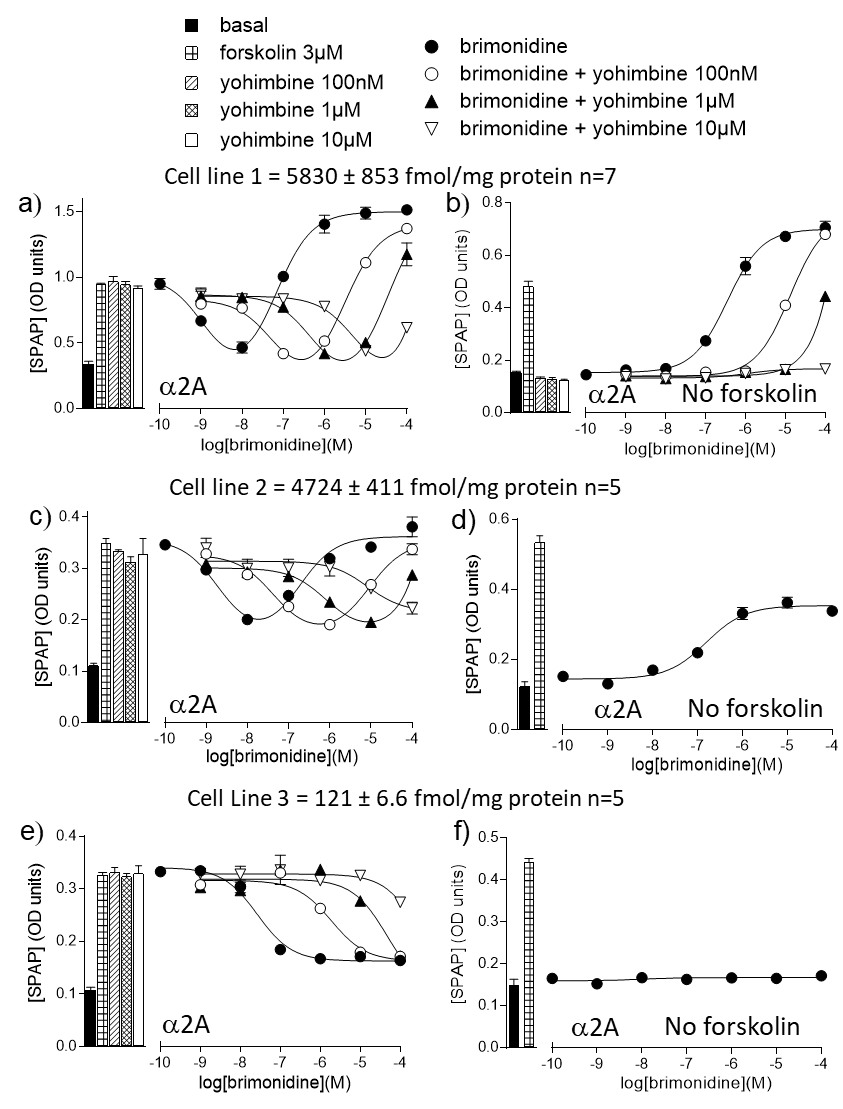


| CHO-α2A | CRE-SPAP (with forskolin) | | | |  | CRE-SPAP (without forskolin) | | |
| --- | --- | --- | --- | --- | --- | --- | --- | --- |
|  | Log IC_50_  (Gi) | Log EC_50_  (Gs) | % inhibition | n |  | Log EC_50_  (Gs) | % 3μM  forskolin | n |
| Cell line 1 α2A receptor expression level = 5830 fmol/mg protein | | | | | | | | |
| brimonidine | -8.94 ± 0.05 | -7.07 ± 0.04 |  | 26 |  | -6.67 ± 0.06 | 160.8 ± 9.6 | 11 |
| para-amino-clonidine | -8.74 ± 0.12 | -6.81 ± 0.15 |  | 8 |  | -6.55 ± 0.10 | 37.6 ± 4.0 | 12 |
| clonidine | -8.18 ± 0.04 | -6.35 ± 0.12 |  | 20 |  |  | <5% | 9 |
| naphazoline | -7.79 ± 0.07 |  | 83.1 ± 3.6 | 16 |  | No response |  | 5 |
|  |  |  |  |  |  |  |  |  |
| Cell line 2 α2A receptor expression level = 4724 fmol/mg protein | | | | | | | | |
| brimonidine | -8.73 ± 0.10 | -6.59 ± 0.10 |  | 12 |  | -6.64 ± 0.12 | 56.1 ± 5.8 | 9 |
| para-amino-clonidine | -8.51 ± 0.26 | -6.29 ± 0.32 |  | 4 |  | -6.84 ± 0.11 | 12.9 ± 4.1 | 4 |
| clonidine | -8.04 ± 0.08 |  | 55.8 ± 2.5 | 17 |  | No response |  | 4 |
| naphazoline | -7.24 ± 0.07 |  | 69.4 ± 5.8 | 3 |  | ND |  |  |
|  |  |  |  |  |  |  |  |  |
| Cell line 3 α2A receptor expression level = 121 fmol/mg protein | | | | | | | | |
| brimonidine | -7.45 ± 0.02 |  | 74.5 ± 1.6 | 12 |  | No response |  | 3 |
| para-amino-clonidine | -7.11 ± 0.18 |  | 67.5 ± 4.8 | 4 |  | No response |  | 4 |
| clonidine | -6.50 ± 0.06 |  | 42.9 ± 3.9 | 12 |  | No response |  | 4 |
| naphazoline | -6.38 ± 0.42 |  | 24.8 ± 4.3 | 3 |  | ND |  |  |

ND – not determined

CRE-SPAP production in CHO-α2A cells in response to brimonidine in the absence and presence of yohimbine in 3 cell lines with different levels of α2A-adrenoceptor expression. a), c) and e) are in the presence of 3 μM forskolin and b), d) and f) in the absence of forskolin. Cell line 1 is from the main manuscript. Bars represent basal CRE-SPAP production and that in response to 3 µM forskolin alone. Data points are mean ± sem of triplicate determinations.

Table of data obtained in CHO-α2A cell lines with biphasic log IC_50_ and EC_50_ values from CRE-SPAP production in presence of forskolin, or in the cases of inhibition only, log IC_50_ and % inhibition from the 3 μM forskolin control. Values are mean ± sem of n determinations.

The log K_D_ values for yohimbine are

1. -8.45 ± 0.03 n=15 (Gi) and -8.65 ± 0.04 n=13 (Gs); b) -8.61 ± 0.06 n=14 (Gs)
2. -8.22 ± 0.07 n=9 (Gi) and -8.64 ± 0.06 n=9 (Gs);
3. -8.56 ± 0.07 n=10 (Gi)

Receptor expression levels were determined from Bmax from ^3^H-rauwolscine whole cell binding. For the cell line 1 (main manuscript cell line), the Bmax was determined from ^3^H-rauwolscine saturation binding (Proudman et al., 2022. Pharmacol Res Perspect. 10(2):e00936. doi: 10.1002/prp2.936). For cell lines 2 and 3, as ^3^H-rauwolscine (stereoisomer of yohimbine) had been determined to have the same affinity (K_D_) as its counterpart yohimbine (Proudman et al., 2022), the Bmax was determined from yohimbine competition curves using the equation:

bound ligand = Bmax x [^3^H-rauwolscine]

[^3^H-rauwolsine] + K_D_ yohimbine.

The protein content was determined by the method of Lowry et al., (1951; J. Biol. Chem. 193: 265-275).

Supplementary Figure 2 - dexmedetomidine responses in CHO-α2A cells


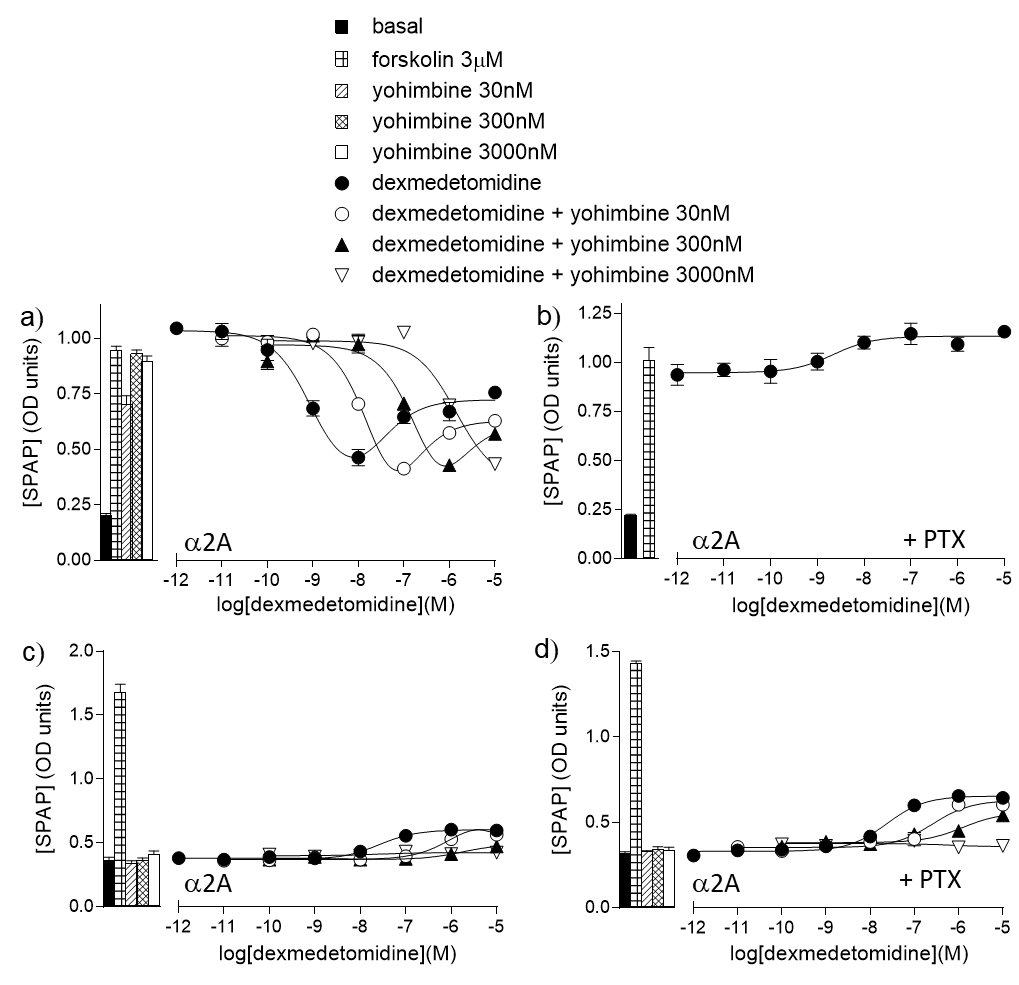


CRE-SPAP in CHO-α2A cells in response to dexmedetomidine in the absence and presence of yohimbine. a) in the presence of 3 μM forskolin, b) in the presence of 3 μM forskolin after 24 hrs PTX pre-treatment, c) in the absence of forskolin and d) in the absence of forskolin after 24 hrs PTX pre-treatment. Bars represent basal CRE-SPAP production, that in response to 3 µM forskolin alone, and that in response to yohimbine 30 nM, 300 nM and 3000 nM alone. Data points are mean ± sem of triplicate determinations.

The log K_D_ values obtained for yohimbine were a) -8.60 ± 0.04, n=17 (Gi), Schild slope 1.11 ± 0.07 n=5, -8.78 ± 0.06, n=10 (Gs); c) -8.77 ± 0.14, n=9 and d) -8.57 ± 0.10, n=13.

Supplementary Figure 3 - moxonidine responses in CHO-α2B cells


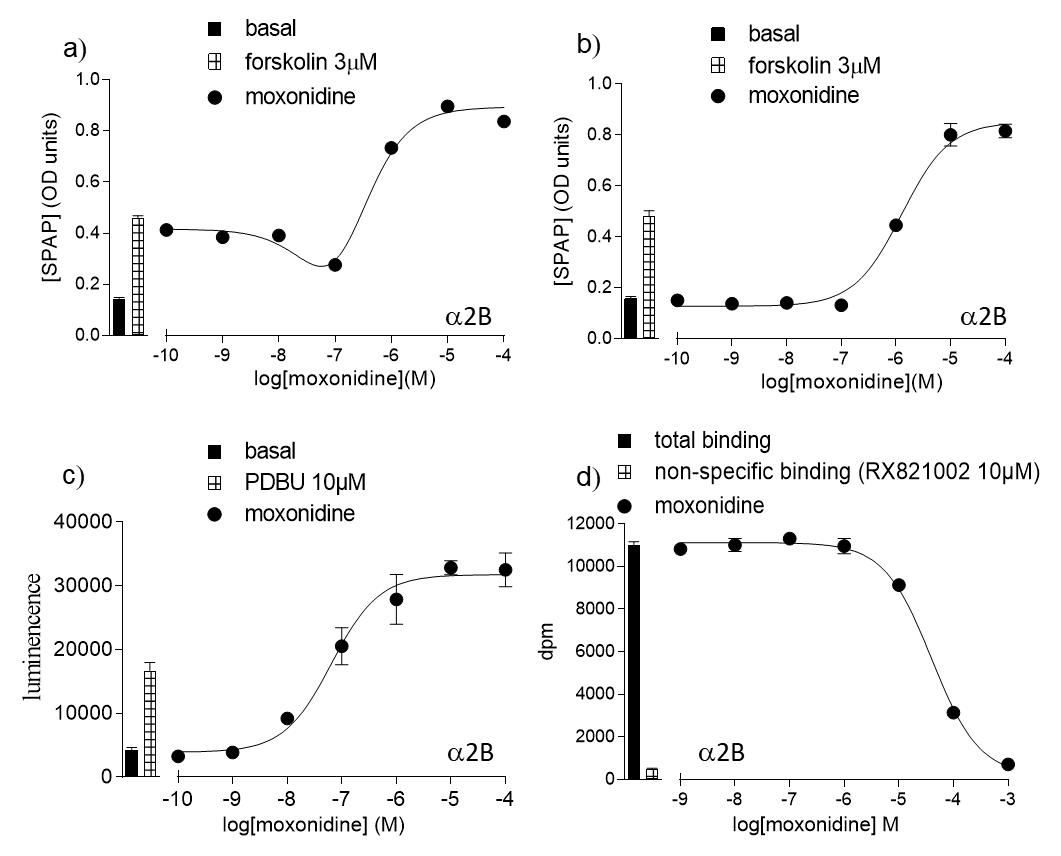


Responses to moxonidine in CHO-α2B cells. a) CRE-SPAP production in the presence of 3 μM forskolin, and b) CRE-SPAP production in the absence of forskolin. Bars represent basal CRE-SPAP production and that in response to 3 µM forskolin alone. c) ERK1/2-phosphorylation. Bars represent basal ERK1/2-phosphorylation and that in response to 10 μM PDBU. and d) inhibition of ^3^H-rauwolscine binding in whole CHO-α2B cells. Bars represent total binding and non-specific binding as determined by 10 μM RX821002. The concentration of ^3^H-rauwolscine in this experiment was 0.60 nM.

Data points are mean ± sem of triplicate determinations.

Supplementary Figure 4 - naphazoline responses in CHO-α2B cells


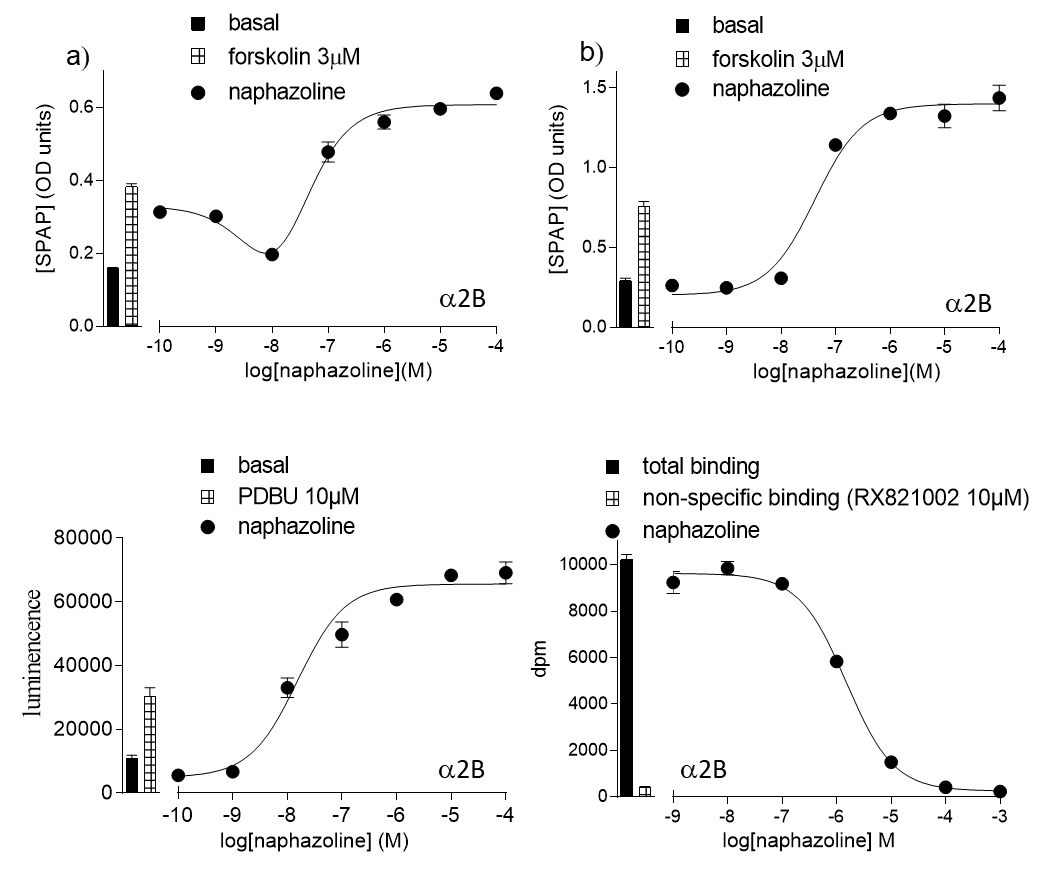


Responses to naphazoline in CHO-α2B cells. a) CRE-SPAP production in the presence of 3 μM forskolin, and b) CRE-SPAP production in the absence of forskolin. Bars represent basal CRE-SPAP production and that in response to 3 µM forskolin alone. c) ERK1/2-phosphorylation. Bars represent basal ERK1/2-phosphorylation and that in response to 10 μM PDBU and d) inhibition of ^3^H-rauwolscine binding in whole CHO-α2B cells. Bars represent total binding and non-specific binding as determined by 10 μM RX821002. The concentration of ^3^H-rauwolscine in this experiment was 0.56 nM.

Data points are mean ± sem of triplicate determinations.

Supplementary Figure 5 - moxonidine responses in CHO-α2C cells


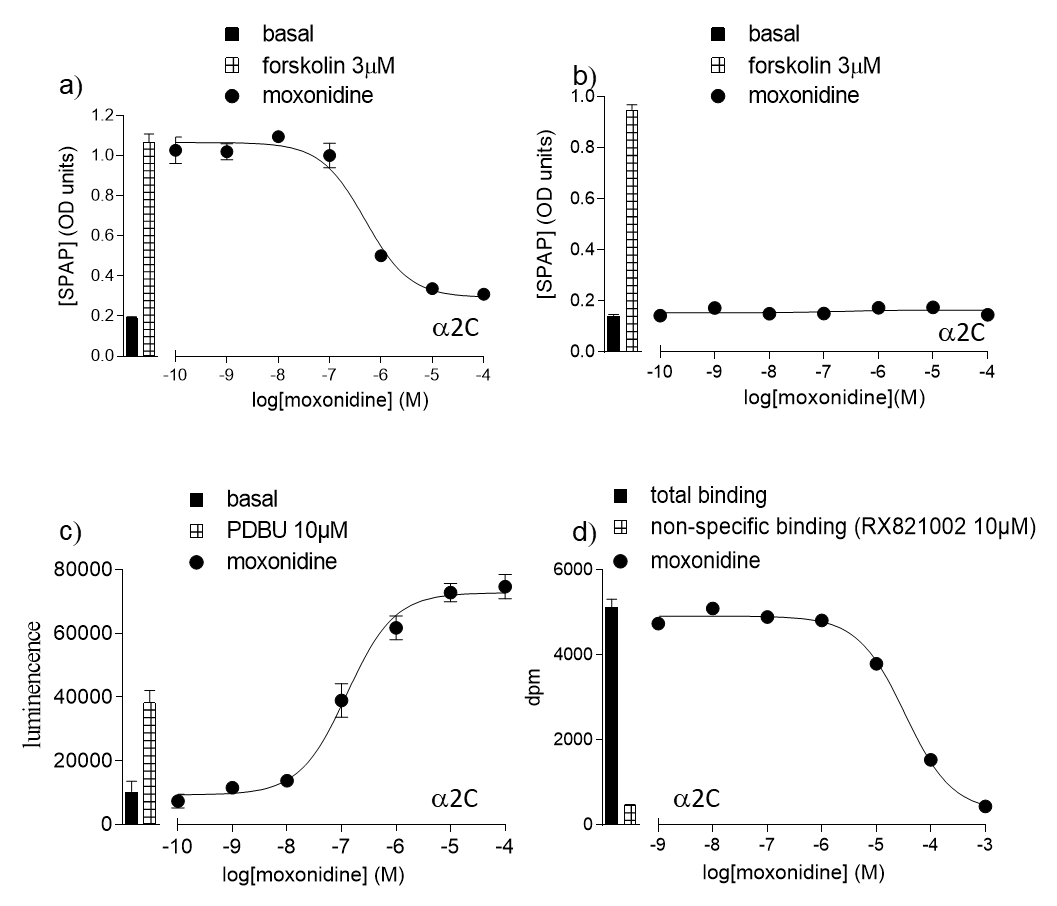


Responses to moxonidine in CHO-α2C cells. a) CRE-SPAP production in the presence of 3 μM forskolin, and b) CRE-SPAP production in the absence of forskolin. Bars represent basal CRE-SPAP production and that in response to 3 µM forskolin alone. c) ERK1/2-phosphorylation. Bars represent basal ERK1/2-phosphorylation and that in response to 10 μM PDBU and d) inhibition of ^3^H-rauwolscine binding in whole CHO-α2C cells. Bars represent total binding and non-specific binding as determined by 10 μM RX821002. The concentration of ^3^H-rauwolscine in this experiment was 0.60 nM.

Data points are mean ± sem of triplicate determinations.

Supplementary Figure 6 - naphazoline responses in CHO-α2C cells


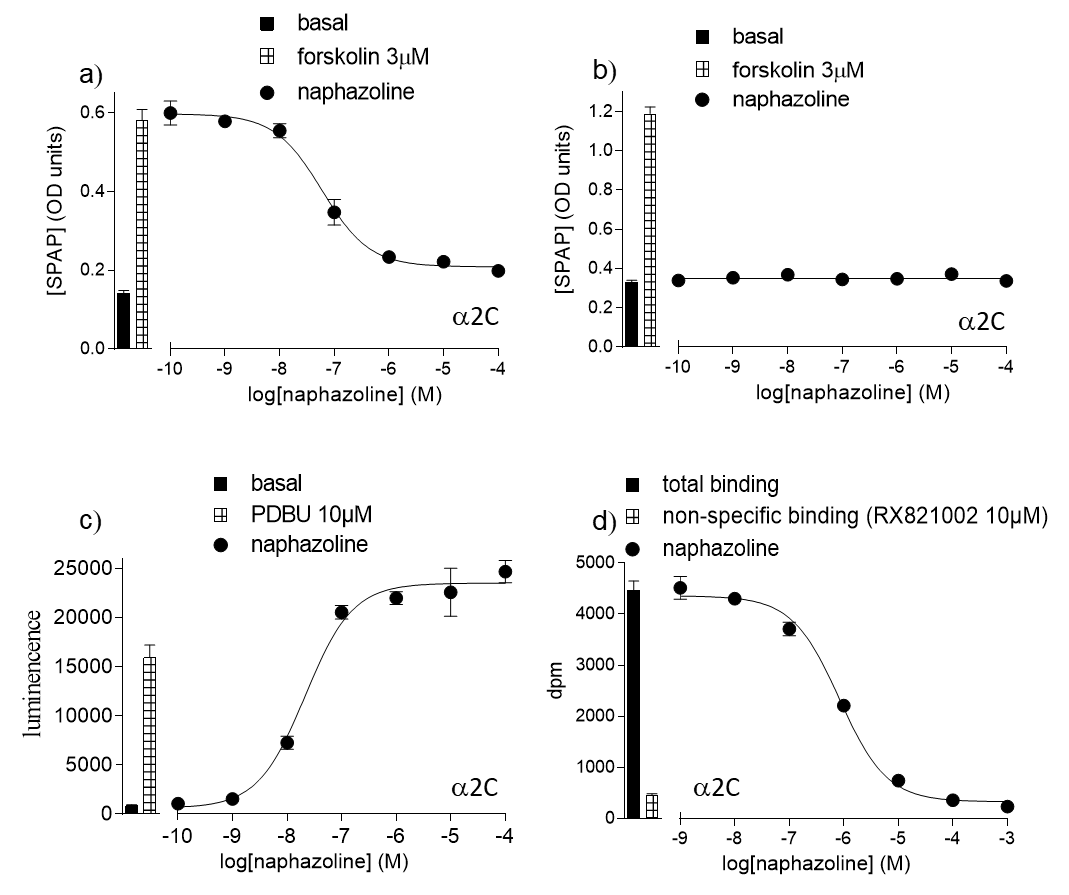


Responses to naphazoline in CHO-α2C cells. a) CRE-SPAP production in the presence of 3 μM forskolin, and b) CRE-SPAP production in the absence of forskolin. Bars represent basal CRE-SPAP production and that in response to 3 µM forskolin alone. c) ERK1/2-phosphorylation. Bars represent basal ERK1/2-phosphorylation and that in response to 10 μM PDBU and d) inhibition of ^3^H-rauwolscine binding in whole CHO-α2C cells. Bars represent total binding and non-specific binding as determined by 10 μM RX821002. The concentration of ^3^H-rauwolscine in this experiment was 0.60 nM.

Data points are mean ± sem of triplicate determinations.

. Supplementary Figure 7 - etilefrine responses in CHO-β1 and CHO-β2 cells


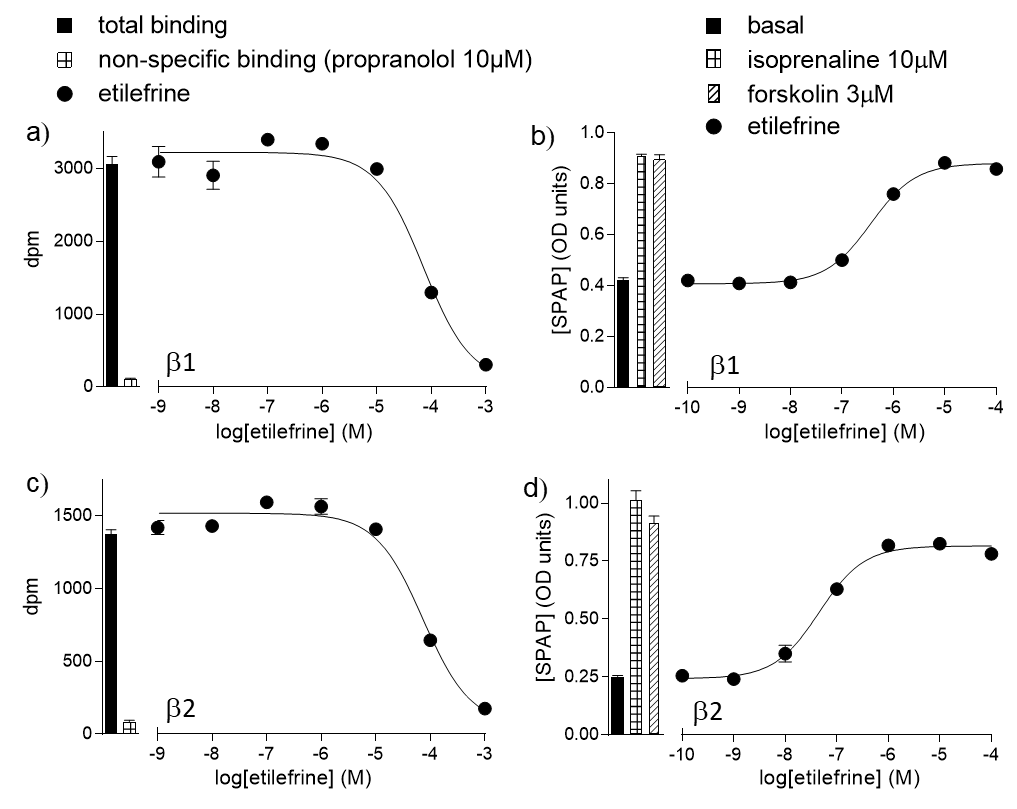


Responses to etilefrine a) and b) CHO-β1 cells and c) and d) CHO-β2 cells.

a) and c) inhibition of ^3^H-CGP12177 whole cell binding. Bars represent total binding and non-specific binding as determined by 10 μM propranolol. The concentration of ^3^H-CGP12177 in these experiments was 0.71 nM.

b and d) CRE-SPAP production in the absence of forskolin. Bars represent basal CRE-SPAP production, that in response to 10 µM isoprenaline or 3 µM forskolin alone.

Data points are mean ± sem of triplicate determinations.

Supplementary table 1 – radioligand binding studies

Ligands (in alphabetical order), with supplier, catalogue number and maximum concentration used in binding assays. The K_D_ values obtained from ^3^H-CGP12177 whole cell binding in CHO-β1 and CHO-β2 cells are also given. Values represent mean ± s.e.m. of n separate experiments. Bromocriptine also had high affinity for α1-subtypes as measured by ^3^H-prazosin whole cell binding: α1A -8.73 ± 0.06 (n=5); α1B -7.96 ± 0.07 (n=5); α1D -7.31 ± 0.15^ep^, n=9.

|  | Supplier and catalogue number | Maximum concentration |  | ^3^H-CGP12177 whole cell binding | | | | |
| --- | --- | --- | --- | --- | --- | --- | --- | --- |
| ligand |  |  |  | CHO-β1 | n |  | CHO-β2 | n |
| A61603 | Tocris – 1052 | 100µM |  | no binding | 5 |  | no binding | 5 |
| adrenaline | Sigma – E4642 | 10mM |  | -4.87 ± 0.06 | 8 |  | -5.97 ± 0.06 | 7 |
| allyphenyline | Sigma – SML1484 | 1mM |  | No binding | 6 |  | No binding | 6 |
| amitraz | Sigma – 45323 | 100μM |  | no binding | 5 |  | no binding | 5 |
| atipamezole | Sigma – A9611 | 30μM |  | IC_50_>-4.5 | 5 |  | IC_50_>-4.5 | 5 |
| BHT920 | Tocris - 2759 | 1mM |  | IC_50_>-3 | 5 |  | IC_50_>-3 | 5 |
| BHT933 | Tocris - 2758 | 1mM |  | no binding | 5 |  | no binding | 5 |
| bromocriptine | Tocris - 0427 | 10µM |  | no binding | 5 |  | -5.85 ± 0.21^ep^ | 5 |
| brimonidine | ARK - AK35795 | 1mM |  | no binding | 5 |  | no binding | 5 |
| buspirone | Sigma – B7148 | 1mM |  | IC_50_>-3 | 5 |  | IC_50_>-3 | 5 |
| chloroethylclonidine | Sigma – B003 | 100μM |  | no binding | 5 |  | no binding | 5 |
| cirazoline | Sigma – C223 | 1mM |  | -4.89 ± 0.08 | 6 |  | -5.26 ± 0.09 | 6 |
| clonidine | Sigma – C7897 | 1mM |  | no binding | 5 |  | no binding | 5 |
| detomidine | Sigma - 34265 | 100μM |  | IC_50_>-4 | 6 |  | IC_50_>-4 | 6 |
| dexmedetomidine | Sigma – SML0956 | 100μM |  | IC_50_>-4 | 5 |  | IC_50_>-4 | 5 |
| dihydroergotamine | Tocris - 0457 | 100μM |  | IC_50_>-4 | 6 |  | -5.25 ± 0.01 | 6 |
| dobutamine | Sigma – D0676 | 1mM |  | -5.36 ± 0.05 | 5 |  | -5.74 ± 0.06 | 5 |
| dopamine | Sigma – H8502 | 10mM |  | -3.57* |  |  | -3.93* |  |
| etilefrine | Sigma - 285749 | 1mM |  | -4.70 ± 0.04 | 5 |  | -4.96 ± 0.04 | 5 |
| ephedrine | ARK – AK390 | 1mM |  | -3.82 ± 0.03^app^ | 5 |  | -4.55 ± 0.04 | 5 |
| fenoterol | Sigma – F1016 | 1mM |  | -5.04* |  |  | -7.03* |  |
| formoterol | Tocris – 1448 | 100μM |  | -6.11* |  |  | -8.63* |  |
| guanabenz | Sigma – G110 | 100μM |  | IC_50_>-4 | 5 |  | IC_50_>-4 | 5 |
| guanfacine | Sigma – G1043 | 100μM |  | IC_50_>-4 | 5 |  | IC_50_>-4 | 5 |
| isoprenaline | Sigma - I5627 | 1mM |  | -6.06* |  |  | -6.64* |  |
| medetomidine | Tocris - 5160 | 100μM |  | no binding | 5 |  | no binding | 5 |
| metaraminol | Sigma – M4778 | 1mM |  | -4.78 ± 0.04 | 5 |  | -4.62 ± 0.03 | 5 |
| methoxamine | Sigma – M6524 | 1mM |  | -4.45 ± 0.09 | 5 |  | -5.22 ± 0.08 | 5 |
| methyldopa | Tocris – 0584 | 100μM |  | No binding | 5 |  | No binding | 5 |
| α-methylnorepinephrine | Sigma – SML0675 | 1mM |  | -5.49 ± 0.05 | 5 |  | -5.00 ± 0.04 | 5 |
| midodrine | Sigma – M8277 | 1mM |  | No binding | 5 |  | No binding | 5 |
| moxonidine | Sellakchem – S2066 | 1mM |  | No binding | 5 |  | No binding | 5 |
| naphazoline | Sigma – 70170 | 1mM |  | IC_50_>-3 | 5 |  | IC_50_>-3 | 5 |
| noradrenaline | Sigma – A0937 | 10mM |  | -5.43 ± 0.03 | 9 |  | -4.74 ± 0.07 | 9 |
| octopamine | Tocris – 2242 | 1mM |  | -3.91* |  |  | -4.03* |  |
| oxymethazoline | Tocris - 1142 | 100μM |  | IC_50_>-4 | 5 |  | IC_50_>-4 | 5 |
| para-amino-clonidine | Sigma – A0779 | 100μM |  | No binding | 5 |  | No binding | 5 |
| R-phenylephrine | Tocris - 2838 | 1mM |  | -4.10 ± 0.09 | 5 |  | -4.66 ± 0.07 | 5 |
| rilmenidine | Tocris - 0790 | 100μM |  | IC_50_>-4 | 5 |  | IC_50_>-4 | 5 |
| RWJ52353 | Tocris - 3935 | 100μM |  | No binding | 5 |  | No binding | 5 |
| salbutamol | Sigma – S5013 | 1mM |  | -4.68* |  |  | -6.01* |  |
| salmeterol | Tocris – 1660 | 100μM |  | -5.73* |  |  | -9.26* |  |
| ST-91 | Tocris – 2638 | 1mM |  | No binding | 5 |  | No binding | 5 |
| synephrine | Sigma – S0752 | 1mM |  | -3.68 ± 0.02^app^ | 5 |  | -4.19 ± 0.05 | 5 |
| T-CG 1000 | Tocris – 5021 | 100μM |  | IC_50_>-4 | 5 |  | IC_50_>-4 | 5 |
| tetrahydrozoline | Sigma – T4264 | 1mM |  | No binding | 5 |  | No binding | 5 |
| tizanidine | Sellakchem – S1437 | 100μM |  | No binding | 5 |  | No binding | 5 |
| UK14304 | Tocris – 0425 | 100μM |  | No binding | 5 |  | No binding | 5 |
| xylazine | Sigma – X1251 | 1mM |  | No binding | 5 |  | No binding | 5 |
| xylometazoline | Sigma – X6000 | 1mM |  | IC_50_~-3.5 | 5 |  | IC_50_>-3 | 5 |

^app^ = apparent affinity. The maximum concentration of competing ligand inhibited most but not all of specific binding. An IC_50_ was determined by extrapolating the curve assuming that all specific binding would be inhibited if a higher concentration of competing ligand were possible.

^ep^ = early plateau, the competing ligand did not fully inhibit specific binding and the inhibition curve reached a plateau of maximal inhibition of binding. The specific binding inhibited by bromocriptine 68.4 ± 1.7% in the CHO-β2 cells and 71.2 ± 3.4% in the CHO-α1D cells

*from Baker (2010). Br. J. Pharmacol. 160: 148-161

Supplementary table 2 – CRE-SPAP functional data

CRE-SPAP responses in CHO-β1 cells, CHO-β2 cells and CHO-CRE-SPAP cells (i.e. parental cell line without any transfected receptor) in alphabetical order of agonist. The maximum concentration used in CRE-SPAP assays is also given. Log EC_50_ values are given (in absence of forskolin) with % of 10 µM isoprenaline maximum response obtained. Ligands were also assessed for inhibitory responses in the presence of 3 µM forskolin – no responses were seen. For ligands that stimulated a response, but the top of the concentration response was not obtained with the highest concentration of agonist, are given as % response at the maximum concentration of agonist used. Values are mean ± sem of n separate experiments.

|  |  | CHO-β1 cells | | | |  | CHO-β2 cells | | | |  | CRE-SPAP cells | | | |
| --- | --- | --- | --- | --- | --- | --- | --- | --- | --- | --- | --- | --- | --- | --- | --- |
| ligand | maximum | Log EC_50_  % 10µM isop | n | Log IC_50_ | n |  | Log EC_50_  % 10µM isop | n | Log IC_50_ | n |  | Log EC_50_  % 10µM isop | n | Log IC_50_ | n |
|  |  | No forskolin |  | With forskolin |  |  | No forskolin |  | With forskolin |  |  | No forskolin |  | With forskolin |  |
| A61603 | 100µM | 9.4 ± 5.0% | 5 | No resp | 5 |  | 48.4 ± 13.0% | 5 | No resp | 5 |  | No resp | 5 | No resp | 5 |
| adrenaline | 100µM | -6.76 ± 0.15  103.7 ± 4.1% | 11 | No resp | 9 |  | -7.43 ± 0.20  101.9 ± 3.5% | 10 | No resp | 8 |  | No resp | 7 | No resp | 7 |
| allyphenyline | 100µM | No resp | 5 | No resp | 5 |  | No resp | 5 | No resp | 5 |  | No resp | 5 | No resp | 5 |
| amitraz | 10µM | No resp | 5 | No resp | 5 |  | No resp | 5 | No resp | 5 |  | No resp | 5 | No resp | 5 |
| atipamezole | 10µM | No resp | 5 | No resp | 5 |  | No resp | 5 | No resp | 5 |  | No resp | 5 | No resp | 5 |
| BHT920 | 100µM | No resp | 5 | No resp | 5 |  | 57.0 ± 5.8% | 5 | No resp | 5 |  | No resp | 5 | No resp | 5 |
| BHT933 | 100µM | No resp | 5 | No resp | 5 |  | No resp | 5 | No resp | 5 |  | No resp | 5 | No resp | 5 |
| bromocriptine | 1µM | No resp | 5 | No resp | 5 |  | No resp | 5 | No resp | 5 |  | No resp | 5 | No resp | 5 |
| brimonidine | 100µM | No resp | 5 | No resp | 5 |  | No resp | 5 | No resp | 5 |  | No resp | 5 | No resp | 5 |
| buspirone | 100µM | No resp | 5 | No resp | 5 |  | No resp | 5 | No resp | 5 |  | No resp | 5 | No resp | 5 |
| chloroethylclonidine | 10µM | No resp | 5 | No resp | 5 |  | No resp | 5 | No resp | 5 |  | No resp | 5 | No resp | 5 |
| cirazoline | 100µM | 9.5 ± 4.7% | 6 | No resp | 5 |  | 15.4 ± 5.2% | 7 | No resp | 7 |  | No resp | 7 | No resp | 7 |
| clonidine | 100µM | No resp | 5 | No resp | 5 |  | No resp | 5 | No resp | 5 |  | No resp | 5 | No resp | 5 |
| detomidine | 10µM | No resp | 5 | No resp | 5 |  | No resp | 5 | No resp | 5 |  | No resp | 5 | No resp | 5 |
| dexmedetonidine | 10µM | No resp | 5 | No resp | 5 |  | No resp | 5 | No resp | 5 |  | No resp | 5 | No resp | 5 |
| dihydroergotamine | 10µM | 11.0 ± 5.3% | 5 | No resp | 6 |  | 16.2 ± 3.9% | 6 | No resp | 6 |  | No resp | 6 | No resp | 6 |
| dobutamine | 100µM | -6.71 ± 0.14  106.4 ± 5.9% | 11 | No resp | 6 |  | -6.56 ± 0.06  100.1 ± 2.4% | 9 | No resp | 6 |  | No resp | 6 | No resp | 6 |
| dopamine | 1mM | -5.44 ± 0.03  105.5 ± 6.0% | 6 | No resp | 6 |  | -5.60 ± 0.08  103.1 ± 7.5% | 6 | No resp | 5 |  | No resp | 6 | No resp | 6 |
| etilefrine | 100µM | -6.53 ± 0.10  94.7 ± 5.3% | 6 | No resp | 6 |  | -7.22 ± 0.06  93.2 ± 5.2% | 6 | No resp | 6 |  | No resp | 6 | No resp | 6 |
| ephedrine | 1mM | -5.21 ± 0.15  50.3 ± 12.7% | 3 | No resp | 3 |  | -6.13 ± 0.07  93.1 ± 3.9% | 7 | No resp | 3 |  | No resp | 3 | No resp | 3 |
| fenoterol | 100µM | -7.72 ± 0.05  109.8 ± 8.8% | 6 | No resp | 6 |  | -9.82 ± 0.06  96.6 ± 5.7% | 6 | No resp | 7 |  | No resp | 7 | No resp | 7 |
| formoterol | 10µM | -8.83 ± 0.15  101.3 ± 4.0 | 9 | No resp | 6 |  | -11.14 ± 0.17  99.3 ± 1.5 | 10 | No resp | 7 |  | No resp | 6 | No resp | 6 |
| guanabenz | 10µM | No resp | 5 | No resp | 5 |  | No resp | 5 | No resp | 5 |  | No resp | 5 | No resp | 5 |
| guanfacine | 100µM | 27.4 ± 7.2% | 5 | No resp | 5 |  | No resp | 5 | No resp | 5 |  | No resp | 5 | No resp | 5 |
| isoprenaline | 100µM | -7.68 ± 0.19  104.0 ± 0.19% | 7 | No resp | 8 |  | -7.90 ± 0.15  99.9 ± 3.0% | 11 | No resp | 8 |  | No resp | 10 | No resp | 10 |
| medetomidine | 10µM | No resp | 5 | No resp | 5 |  | No resp | 5 | No resp | 5 |  | No resp | 5 | No resp | 5 |
| metaraminol | 100µM | -6.37 ± 0.10  98.7 ± 7.6% | 6 | No resp | 6 |  | -6.35 ± 0.08  90.0 ± 5.9% | 6 | No resp | 6 |  | No resp | 6 | No resp | 6 |
| methoxamine | 100µM | No resp | 5 | No resp | 5 |  | No resp | 5 | No resp | 5 |  | No resp | 5 | No resp | 5 |
| methyldopa | 10µM | No resp | 5 | No resp | 5 |  | No resp | 5 | No resp | 5 |  | No resp | 5 | No resp | 5 |
| α-methylnorepin  ephrine | 100µM | -7.06 ± 0.17  107.5 ± 4.6% | 10 | No resp | 6 |  | -6.86 ± 0.11  107.1 ± 2.3% | 6 | No resp | 6 |  | No resp | 6 | No resp | 6 |
| midodrine | 100µM | No resp | 5 | No resp | 5 |  | No resp | 5 | No resp | 5 |  | No resp | 5 | No resp | 5 |
| moxonidine | 100µM | No resp | 5 | No resp | 5 |  | No resp | 5 | No resp | 5 |  | No resp | 5 | No resp | 5 |
| naphazoline | 100µM | No resp | 7 | No resp | 5 |  | No resp | 7 | No resp | 6 |  | No resp | 6 | No resp | 6 |
| noradrenaline | 100µM | -7.13 ± 0.23  109.7 ± 4.8% | 11 | No resp | 9 |  | -6.66 ± 0.13  101.9 ± 3.5 | 10 | No resp | 5 |  | No resp | 5 | No resp | 5 |
| octopamine | 1mM | -5.70 ± 0.10  101.9 ± 6.5% | 7 | No resp | 6 |  | -5.20 ± 0.04  68.4 ± 5.4% | 7 | No resp | 6 |  | No resp | 6 | No resp | 6 |
| oxymethazoline | 100µM | No resp | 6 | No resp | 5 |  | No resp | 5 | No resp | 5 |  | No resp | 5 | No resp | 5 |
| para-amino-clonidine | 10µM | No resp | 6 | No resp | 6 |  | No resp | 6 | No resp | 6 |  | No resp | 6 | No resp | 6 |
| R-phenylephrine | 100µM | -5.84 ± 0.16  89.7 ± 7.8% | 8 | No resp | 6 |  | -7.13 ± 0.07  98.9 ± 3.2% | 9 | No resp | 6 |  | No resp | 6 | No resp | 6 |
| rilmenidine | 10µM | No resp | 5 | No resp | 5 |  | No resp | 5 | No resp | 5 |  | No resp | 5 | No resp | 5 |
| RWJ52353 | 10µM | No resp | 5 | No resp | 5 |  | No resp | 5 | No resp | 5 |  | No resp | 5 | No resp | 5 |
| salbutamol | 100µM | -6.45 ± 0.06  103.7 ± 10.2% | 5 | No resp | 5 |  | -8.80 ± 0.13  100.5 ± 5.0% | 7 | No resp | 6 |  | No resp | 7 | No resp | 7 |
| ST-91 | 100µM | No resp | 5 | No resp | 5 |  | No resp | 6 | No resp | 6 |  | No resp | 6 | No resp | 6 |
| synephrine | 100µM | -5.29 ± 0.14  95.0 ± 2.1% | 8 | No resp | 5 |  | -6.21 ± 0.07  97.3 ± 6.1% |  | No resp | 6 |  | No resp | 6 | No resp | 6 |
| T-CG 1000 | 10µM | No resp | 5 | No resp | 5 |  | No resp | 5 | No resp | 5 |  | No resp | 5 | No resp | 5 |
| tetrahydrozoline | 100µM | No resp | 5 | No resp | 5 |  | No resp | 6 | No resp | 6 |  | No resp | 6 | No resp | 6 |
| tizanidine | 100µM | No resp | 5 | No resp | 5 |  | No resp | 5 | No resp | 5 |  | No resp | 5 | No resp | 5 |
| UK14304 | 10µM | No resp | 5 | No resp | 5 |  | 9.2 ± 3.8% | 5 | No resp | 5 |  | No resp | 5 | No resp | 5 |
| xylazine | 100µM | No resp | 5 | No resp | 5 |  | No resp | 5 | No resp | 5 |  | No resp | 5 | No resp | 5 |
| xylometazoline | 100µM | No resp | 5 | No resp | 5 |  | No resp | 5 | No resp | 5 |  | No resp | 5 | No resp | 5 |

No resp = no response
